# NLRP3 licenses NLRP11 for inflammasome activation in human macrophages

**DOI:** 10.1038/s41590-022-01220-3

**Published:** 2022-05-27

**Authors:** Anu Gangopadhyay, Savita Devi, Shivendra Tenguria, Jessica Carriere, Huyen Nguyen, Elisabeth Jäger, Hemisha Khatri, Lan H. Chu, Rojo A. Ratsimandresy, Andrea Dorfleutner, Christian Stehlik

**Affiliations:** 1 Department of Academic Pathology, Cedars Sinai Medical Center, Los Angeles, CA USA; 2 Driskill Graduate Program in Life Sciences, Feinberg School of Medicine, Northwestern University, Chicago, IL USA; 3 Department of Biomedical Sciences, Cedars Sinai Medical Center, Los Angeles, CA USA; 4 Samuel Oschin Comprehensive Cancer Institute, Cedars Sinai Medical Center, Los Angeles, CA USA; 5 Present Address: Synthekine, Menlo Park, CA USA; 6 Present Address: Department of Immunology, University of Washington, Seattle, WA USA; 7 Present Address: Department of Immunology, Genentech, South San Francisco, CA USA

**Keywords:** Inflammasome, NOD-like receptors

## Abstract

Intracellular sensing of stress and danger signals initiates inflammatory innate immune responses by triggering inflammasome assembly, caspase-1 activation and pyroptotic cell death as well as the release of interleukin 1β (IL-1β), IL-18 and danger signals. NLRP3 broadly senses infectious patterns and sterile danger signals, resulting in the tightly coordinated and regulated assembly of the NLRP3 inflammasome, but the precise mechanisms are incompletely understood. Here, we identified NLRP11 as an essential component of the NLRP3 inflammasome in human macrophages. NLRP11 interacted with NLRP3 and ASC, and deletion of NLRP11 specifically prevented NLRP3 inflammasome activation by preventing inflammasome assembly, NLRP3 and ASC polymerization, caspase-1 activation, pyroptosis and cytokine release but did not affect other inflammasomes. Restored expression of NLRP11, but not NLRP11 lacking the PYRIN domain (PYD), restored inflammasome activation. NLRP11 was also necessary for inflammasome responses driven by NLRP3 mutations that cause cryopyrin-associated periodic syndrome (CAPS). Because NLRP11 is not expressed in mice, our observations emphasize the specific complexity of inflammasome regulation in humans.

## Abstract (web summary)

Stehlik and colleagues show that NLRP11 is an essential component of the NLRP3 inflammasome in human macrophages.

## Main

Germline-encoded, cytosolic pattern recognition receptors sense infectious and sterile stress signals and play a key role in mounting an inflammatory response that eradicates infections and facilitates wound healing and homeostasis. A consequence of intracellular pattern recognition is the activation of caspase-1 within the inflammasome^1,2^, and NOD-like receptor (NLR) family pyrin domain containing 3 (NLRP3) is a prominent inflammasome sensor of microbial patterns, self-derived danger signals and environmental cues^3–5^. Excessive or mutation-driven NLRP3 responses cause a wide range of inflammatory diseases^6^, including CAPS, which is caused by gain-of-function mutations in *NLRP3* (ref. ^7^). NLRP3 consists of an N-terminal PYD, a central NAIP [neuronal apoptosis inhibitor protein], C2TA [class 2 transcription activator, of the MHC], HET-E [heterokaryon incompatibility] and TP1 [telomerase-associated protein 1] (NACHT) domain and C-terminal leucine-rich regions (LRRs). The NACHT has ATPase activity and is bound to the LRRs and/or the PYD to maintain an inactive conformation^8,9^. Once this autoinhibition is released, NLRP3 oligomerizes, and its PYD nucleates polymerization of the adaptor protein ASC, which serves as an amplification mechanism and proceeds in a prion-like, self-perpetuating manner, establishing a temporal-spatial threshold control^10–12^. Polymerized ASC filaments eventually assemble into the characteristic single macromolecular aggregate (speck)^13,14^. ASC polymerization in turn nucleates caspase-1 polymerization by caspase recruitment domain (CARD)–CARD interactions, resulting in its induced, proximity-mediated activation^15^. Caspase-1 is ultimately responsible for the induction of pyroptosis through the cleavage of gasdermin D (GSDMD) and subsequent GSDMD pore formation, maturation and release of the proinflammatory cytokines IL-1β and IL-18 and the release of danger signals, including IL-1α, HMGB1 and polymerized ASC particles^16–18^. NLRP3 inflammasome activation proceeds in two steps. Priming includes the upregulation of inflammasome components, including NLRP3 and the substrate IL-1β, a metabolic shift from oxidative phosphorylation to glycolysis and the post-translational modifications of NLRP3, ASC and caspase-1 (refs. ^3–5^). NLRP3 is activated by diverse stimuli^3^, and K^+^ efflux has been proposed as the unifying mechanism for NLRP3 activation^19^. Protein oligomerization is a common mechanism for the activation of innate immune signaling and NLRP3 oligomerization, and particularly the downstream ASC polymerization, are key events in inflammasome activation^10,11^. Among the NLRP3 regulatory proteins, NEK7 promotes inflammasome activation by bridging two NLRP3 molecules, which is insufficient to induce NLRP3 oligomerization^20–23^. GBP5 enables NLRP3–ASC binding in response to soluble, but not crystalline, agonists^24^, implying that other crucial, yet-unknown cofactors are necessary for NLRP3 oligomerization, inflammasome assembly and activation. To date, the precise mechanism, especially in humans, remains unclear.

Here, we report the identification of NLRP11 as an NLRP3 inflammasome component in human macrophages. NLRP11 bound to ASC and NLRP3 and was required for NLRP3 oligomerization and ASC polymerization. In the absence of NLRP11, NLRP3-mediated caspase-1 activation and release of IL-1β and IL-18 were defective, but activation of AIM2, NLRC4 and NLRP7 inflammasomes was not affected. The NLRP3–ASC–NLRP11 complex only assembled after NLRP3 inflammasome activation, which required NLRP11^PYD^. NLRP11 was also necessary for the release of IL-1β induced by the CAPS-associated NLRP3 mutant NLRP3^R260W^, which placed NLRP11 at an essential step in human NLRP3 inflammasome assembly and activation. Our study therefore provides important insights into NLRP3 inflammasome regulation in human macrophages.

## Results

### NLRP11 is required for NLRP3-mediated cytokine release

**Fig. 1:**
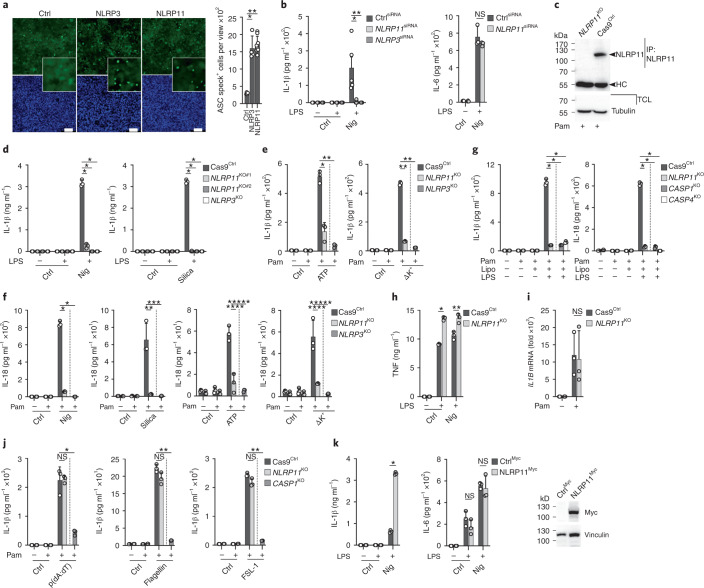
NLRP11 is required for NLRP3-mediated cytokine release. **a**, Fluorescence microscopy of EGFP (green) and 4,6-diamidino-2-phenylindole (DAPI) (blue) in HEK293^ASC-EGFP^ cells transiently transfected with empty plasmid (Ctrl), NLRP3 or NLRP11 (left) and quantification of ASC speck^+^ cells/view (right) (Ctrl: *n* = 4, NLRP3: *n* = 4, NLRP11: *n* = 8; mean ± standard deviation (s.d.)); *P = 0.0003, **P < 0.0001. scale bars, 100 μm. **b**, IL-1β and IL-6 enzyme-linked immunosorbent assay (ELISA) of cleared culture supernatant (SN) from human macrophages transfected with Ctrl^siRNA^, *NLRP11*^siRNA^ or *NLRP3*^siRNA;^ left untreated; primed with LPS (200 ng ml^−1^, 4 h); and primed and activated with nigericin (Nig) (5 μM, 30 min) (IL-1β: *n* = 5, IL-6: *n* = 3, mean ± s.d.); *P = 0.0043, **P = 0.0079. **c**, Immunoprecipitation (IP) with immobilized anti-NLRP11 antibodies using total cell lysates (TCLs) from Cas9^Ctrl^ and *NLRP11*^KO^ THP-1 cells primed with Pam3CSK4 (Pam) (1 μg ml^−1^, 4 h) and analysis by immunoblot alongside TCL for NLRP11 and tubulin loading control. The arrowheads mark NLRP11 and immunoglobulin G heavy chain (HC). **d**–**f**, IL-1β (d,e) and IL-18 (f) ELISA of SN from Cas9^Ctrl^, *NLRP11*^KO#1^, *NLRP11*^KO#2^ and *NLRP3*^KO^ cells left untreated, primed with LPS (200 ng ml^−1^, 4 h) or Pam3CSK4 (1 μg ml^−1^, 4 h) and primed and activated with nigericin (5 μM, 30 min), silica (200 μg ml^−1^, 6 h), ATP (5 mM, 25 min) or cultured in K^+^-free medium (3 h) (*n* = 3, mean ± s.d.). *P < 0.0001 (d), *P = 0.0009, **P < 0.0001 (e); *P < 0.0001, **P = 0.0044, ***P = 0.0039, ****P = 0.002, *****P = 0.0002, ******P = 0.0077, *******P = 0.0037 (f). The dotted line indicates that for *NLRP3*^KO^ only the Pam3CSK4 + nigericin group is shown as control. **g**, IL-1β and IL-18 ELISA of SN from Cas9^Ctrl^, *NLRP11*^KO^, *CASP1*^KO^ or *CASP4*^KO^ cells left untreated, primed with Pam3CSK4 (1 μg ml^−1^, 4 h) and Lipofectamine 2000 (Lipo) transfected with or without LPS (1 μg ml^−1^, 4 h) (*n* = 3, mean ± s.d.); *P < 0.0001. The dotted line indicates that for *CASP1*^KO^ and *CASP4*^KO^, only the Pam3CSK4 + LPS group is shown as control. **h**, TNF ELISA of SNs from Cas9^Ctrl^ and *NLRP11*^KO^ cells left untreated, primed with LPS (200 ng ml^−1^, 4 h) and primed and activated with nigericin (5 μM, 30 min) (*n* = 3, mean ± s.d.); *P < 0.0001, **P = 0.0074. **i**, Quantitative real-time PCR (qPCR) of *IL1B* mRNA from Cas9^Ctrl^ and *NLRP11*^KO^ cells left untreated or primed with Pam3CSK4 (1 μg ml^−1^, 2 h) is presented as fold change compared to control cells (*n* = 3, mean ± s.d.). **j**, IL-1β ELISA of SN from Cas9^Ctrl^, *NLRP11*^KO^ and *CASP1*^KO^ cells left untreated, primed with Pam3CSK4 (1 μg ml^−1^, 2 h) and primed and transfected with poly(dA:dT) (1 μg ml^−1^, 4 h), flagellin (0.5 μg ml^−1^, 4 h) or FSL-1 (0.2 μg ml^−1^, 4 h) (*n* = 3, mean ± s.d.); *P = 0.0025; **P < 0.0001. The dotted line indicates that for *CASP1*^KO^, only the Pam3CSK4 + poly(dA:dT), flagellin and FSL-1 transfected groups are shown as control. **k**, IL-1β and IL-6 ELISA of SN from Ctrl^Myc^ and NLRP11^Myc^ THP-1 cells left untreated, primed with LPS (200 ng ml^−1^, 4 h) and primed and activated with nigericin (5 μM, 30 min) (*n* = 3, mean ± s.d.); *P < 0.0001. Immunoblot of TCLs for Myc and vinculin loading control. NS, not significant. Source data

**Extended Data Fig. 1:**
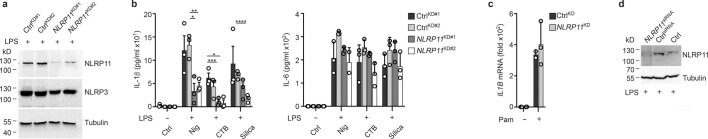
Knockdown of *NLRP11* impairs NLRP3-mediated cytokine release. **(a)** Immunoblot for NLRP11, NLRP3 and tubulin loading control using TCL of LPS-primed (200 ng/ml, 4 h) stable control (Ctrl^KD#1^ and Ctrl^KD#2^) and *NLRP11* knockdown (KD) THP-1 cells (*NLRP11*^KD#1^ and *NLRP11*^KD#2^). **(b)** IL-1β and IL-6 ELISA of SN from Ctrl^KD#1^, Ctrl^KD#2^, *NLRP11*^KD#1^ and *NLRP11*^KD#2^ cells left untreated or primed with LPS (200 ng ml^−1^, 4 h) and primed+activated with Nig (5 µM, 30 min), CTB (20 µg ml^−1^, 6 h) and Silica (200 µg ml^−1^, 6 h); (n = 3, mean ± s.d.); *p = 0.0121; **p = 0.0068, ***p = 0.0101, ****p = 0.0018. **(c)** Quantitative real-time PCR of *IL1B* mRNA levels from Ctrl^KD^ and *NLRP11*^KD^ cells left untreated and primed with Pam3CSK4 (1 μg ml^−1^, 2 h) and presented as fold induction compared to Ctrl cells (n = 3, mean ± s.d.). **(d)** Immunoblot for NLRP11 and tubulin loading control using TCL of primary human macrophages either not transfected (Ctrl), transfected with Ctrl^siRNA^ or *NLRP11*^siRNA^ and primed with LPS (200 ng/ml, 4 h). Source data

**Extended Data Fig. 2:**
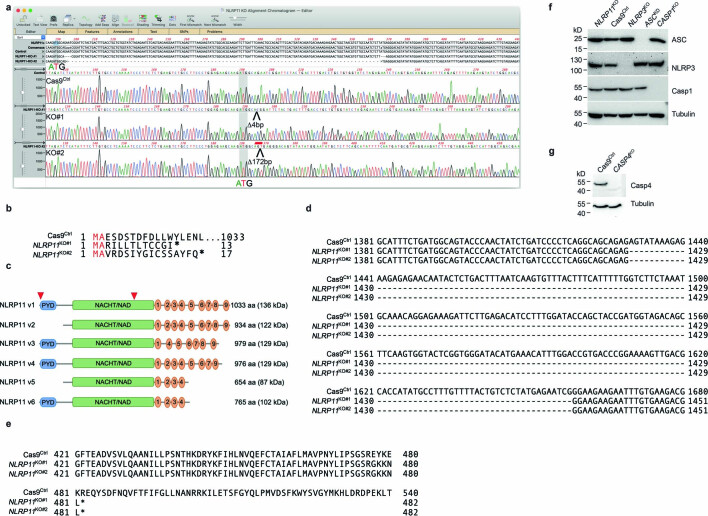
*NLRP11* knockout THP-1 cells. **(a)** PCR amplification and sequencing analysis of Cas9^Ctrl^, *NLRP11*^KO#1^ and *NLRP11*^KO#2^ THP-1 cells, showing two distinct deletions of 4 and 172 bp. **(b)** The predicted amino acid sequence and protein length of the resulting NLRP11 proteins are indicated, with the premature stop marked by an asterisk. **(c)** Domain architecture of the six predicted NLRP11 isoforms. A red arrowhead marks the 2 gRNA targeting sites. **(d)** PCR amplification and sequence analysis of Cas9^Ctrl^, *NLRP11*^KO#1^ and *NLRP11*^KO#2^ cells showing the 229 bp deletion in the NACHT/NAD. **(e)** The predicted amino acid sequence and protein length of the resulting NLRP11 proteins are indicated, with the premature stop marked by an asterisk. **(f,g)** Immunoblot for **(f)** ASC, NLRP3, Caspase-1 and tubulin loading control using TCL from Cas9^Ctrl^, *NLRP11*^KO#1^, *NLRP3*^KO^, *ASC*^KD^ and *CASP1*^KO^ cells and **(g)** caspase-4 and tubulin loading control from TCL of Cas9^Ctrl^ and *CASP4*^KO^ cells. Source data

**Extended Data Fig. 3:**
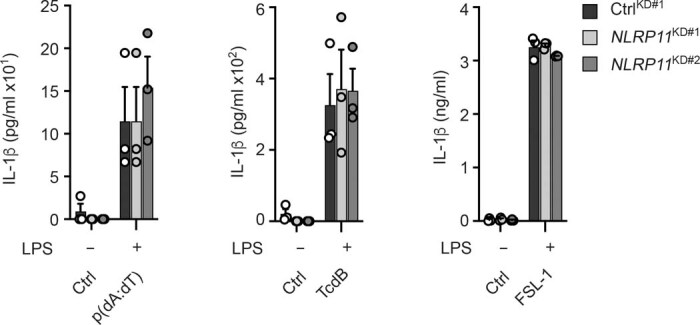
Knockdown of *NLRP11* does not impair IL-1β release by non-NLRP3 inflammasomes. IL-1β ELISA of SN from Ctrl^KD^ and *NLRP11*^KD^ cells left untreated or primed with LPS (200 ng ml^−1^, 4 h) and primed+activated with TcdB (10 μg ml^−1^, 8 h), transfected with poly(dA:dT) (1 μg ml^−1^, 4 h) or FSL-1 (0.2 μg ml^−1^, 4 h); (n = 3, mean ± s.d.). Source data

ASC polymerization is nucleated by PYD–PYD interactions between the inflammasome sensors and ASC^10,11^. To identify NLRs that can nucleate ASC polymerization, we transfected NLRs into HEK293^ASC-EGFP^ cells, which stably express diffusely localized ASC-EGFP. Transfection of NLRP3 and NLRP11, but not empty plasmid (Ctrl), similarly promoted the formation of speck-like aggregates, indicating ASC polymerization (Fig. 1a). Next, we generated stable human THP-1 monocytic cells in which NLRP11 expression was knocked down (*NLRP11*^KD^) with two different short hairpin RNAs (shRNAs) (Extended Data Fig. 1a) and determined the inflammasome-mediated release of IL-1β in response to the NLRP3 activators, silica, nigericin and cholera toxin B (CTB) by ELISA (Extended Data Fig. 1b). IL-1β release was strongly impaired in both *NLRP11*^KD^ cells compared to two non-targeting shRNA control (Ctrl^KD^) cells (Extended Data Fig. 1b). IL-6 is secreted independently of inflammasome activation and was not affected (Extended Data Fig. 1b). Reduced IL-1β release was not a result of impaired transcription of *IL1B*, as indicated by quantitative real-time polymerase chain reaction (PCR) (Extended Data Fig. 1c). Moreover, release of IL-1β, but not IL-6, was reduced in primary human macrophages with short interfering RNA (siRNA) silenced *NLRP11* (*NLRP11*^siRNA^) compared to control siRNA (Ctrl^siRNA^) (Fig. 1b and Extended Data Fig. 1d), indicating NLRP11 contributed to the efficient activation of the NLRP3 inflammasome. Because *NLRP11* knock down and silencing did not completely abolish NLRP11 expression, we used CRISPR-Cas9 to knock out *NLRP11* in THP-1 cells. Sequencing of two independent clones (*NLRP11*^KO#1^ *NLRP11*^KO#2^) indicated a deletion of 4 bp and 172 bp adjacent to the start ATG (Extended Data Fig. 2a), resulting in a frame shift and the introduction of a premature stop codon after amino acid 13 or 17, respectively (Extended Data Fig. 2b). To prevent the expression of various splice forms predicted for NLRP11 (Extended Data Fig. 2c), including the ones using an alternative start site downstream of the PYD, we additionally used CRISPR-Cas9 to target the NACHT/NAD in *NLRP11*^KO#1^ and *NLRP11*^KO#2^ cells. A 229-bp deletion caused a frame shift (Extended Data Fig. 2d) and premature stop (Extended Data Fig. 2e) in both cell lines leading to complete loss of NLRP11 expression (Fig. 1c), without impacting the expression of NLRP3, ASC or caspase-1 (Extended Data Fig. 2f). THP-1 cells with an shRNA-mediated knockdown of *ASC* (*ASC*^KD^) and THP-1 cells with CRISPR-Cas9-generated deletion of *NLRP3* (*NLRP3*^KO^), *CASP1* (*CASP1*^KO^), *CASP4* (*CASP4*^KO^) and Cas9^Ctrl^ cells were used as controls (Extended Data Fig. 2f,g)^13,25^. Activation of primed *NLRP11*^KO^ cells with soluble (nigericin, ATP) and crystalline (silica) NLRP3 activators, as well as K^+^ efflux, resulted in loss of IL-1β (Fig. 1d,e) and IL-18 (Fig. 1f) release compared to Cas9^Ctrl^ cells. IL-1β and IL-18 release was also lost upon noncanonical activation of the NLRP3 inflammasome following transfection of lipopolysaccharide (LPS) directly into the cytosol of primed *NLRP11*^KO^ cells, similar to both *CASP1*^KO^ and *CASP4*^KO^ cells (Fig. 1g). Primed and nigericin-activated *NLRP11*^KO^ cells had unaltered secretion of the inflammasome-independent tumor necrosis factor (TNF) (Fig. 1h) and unaltered *IL1B* transcription (Fig. 1i) compared to Cas9^Ctrl^ cells. In agreement with reports that NLRP11 negatively regulates NF-κB signaling^26,27^, *NLRP11*^KO^ cells showed a slight increase in TNF release compared to Cas9^Ctrl^ cells. NLRP11 selectively affected IL-1β release by the NLRP3 inflammasome but not by AIM2, NLRC4 and NLRP7 inflammasomes, activated with poly(dA:dT), flagellin and FSL-1 transfection, respectively (Fig. 1j). *NLRP11*^KD^ also did not affect IL-1β release induced by transfection with poly(dA:dT), *Clostridium difficile* toxin B (TcdB) and FSL-1 for AIM2, pyrin and NLRP7 inflammasome activation, respectively (Extended Data Fig. 3). Furthermore, the stable expression of Myc-tagged NLRP11 in THP-1 cells (NLRP11^Myc^) resulted in increased IL-1β release in response to nigericin compared to Myc control (Ctrl^Myc^) cells, without affecting the release of IL-6 (Fig. 1k). Collectively, these data strongly indicated that NLRP11 was required for NLRP3-mediated IL-1β and IL-18 release in human macrophages.

### NLRP11 is required for NLRP3-mediated caspase-1 activation

**Fig. 2:**
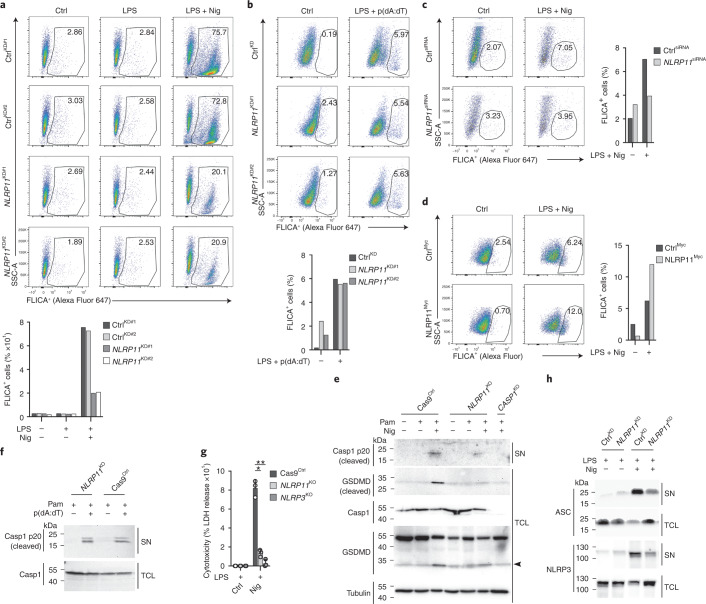
NLRP11 is required for NLRP3-mediated caspase-1 activation. **a**–**d**, Flow cytometry of fluorochrome-labeled inhibitors of caspases assay (FLICA) signals plotted versus side scatter area (SSC-A) in Ctrl^KD^ and *NLRP11*^KD^ cells (a,b) and primary human macrophages transfected with Ctrl^siRNA^ or *NLRP11*^siRNA^ (c) or Ctrl^Myc^ and NLRP11^Myc^ cells left untreated, primed with LPS (1 μg ml^−1^, 1 h) and primed and activated with nigericin (5 μM, 45 min) (a,c), nigericin (5 μM, 15 min) (d) or transfected with poly(dA:dT) (6 ng ml^−1^, 4 h) (b). **e**, Immunoblot for cleaved and total caspase-1, cleaved and total GSDMD and tubulin loading control from SN and TCL of Cas9^Ctrl^, *NLRP11*^KO^ and *CASP1*^KO^ cells left untreated, primed with Pam3CSK4 (1 μg ml^−1^, 4 h) and primed and activated with nigericin (5 μM, 30 min). The arrowhead marks the cleaved GSDMD fragment also detected by the total GSDMD antibody. **f**, Immunoblot for cleaved and total caspase-1 using SNs and TCLs of Cas9^Ctrl^ and *NLRP11*^KO^ cells left untreated, primed with Pam3CSK4 (1 μg ml^−1^, 2 h) and primed + transfected with poly(dA:dT) (2 μg ml^−1^, 6 h). **g**, LDH release from Cas9^Ctrl^, *NLRP11*^KO^ and *NLRP3*^KO^ cells primed with LPS (200 ng ml^−1^, 4 h) or primed and activated with nigericin (5 μM, 30 min) is presented as percent cytotoxicity compared to maximum LDH release (*n* = 3, mean ± s.d.); *P = 0.0002, **P = 0.0001. **h**, Immunoblot for ASC and NLRP3 using SN and TCL of Ctrl^KD^ and *NLRP11*^KD^ cells primed with LPS (200 ng ml^−1^, 4 h) or primed and activated with nigericin (5 μM, 15 min). Source data

**Extended Data Fig. 4:**
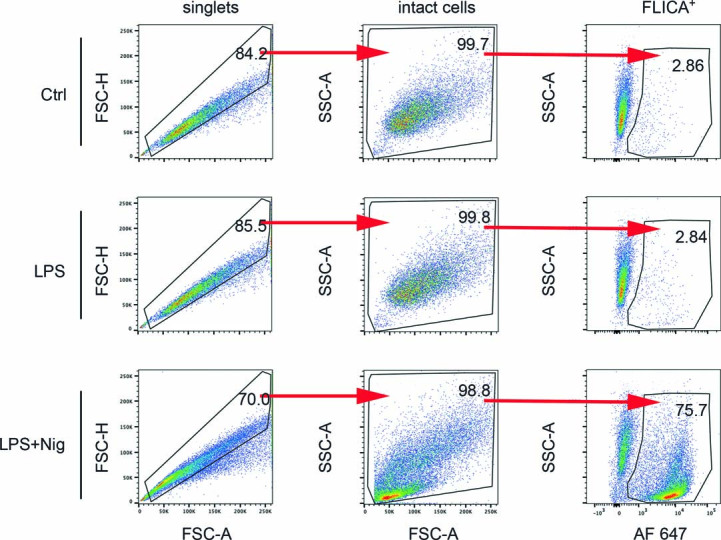
FLICA gating strategy. Gating strategy used for the FLICA in Fig. 2a-d of Ctrl^KD#1^ cells left untreated, primed with LPS (1 μg ml^−1^, 1 h) and primed+activated with Nig (5 μM, 45 min). Total cells were gated for singlets, intact cells and for Alexa Fluor (AF) 647^+^ cells. All samples were gated following this strategy.

Next, we directly assessed caspase-1 activity in Ctrl^KD^ cells, which showed robust activation of caspase-1 in response to nigericin (Fig. 2a) and poly(dA:dT) (Fig. 2b), whereas *NLRP11*^KD^ cells showed markedly reduced caspase-1 activation in response to nigericin (Fig. 2a and Extended Data Fig. 4), but not to poly(dA:dT) (Fig. 2b). Nigericin-mediated caspase-1 activation was also reduced in *NLRP11*^siRNA^ transfected human macrophages compared to Ctrl^siRNA^ transfected cells (Fig. 2c), whereas nigericin-mediated caspase-1 activation was enhanced in NLRP11^Myc^ cells compared to Ctrl^Myc^ cells (Fig. 2d). Accordingly, the release of the cleaved, p20 form of active caspase-1 was diminished, and the subsequent proteolytic cleavage of the caspase-1 substrate GSDMD was abolished in primed *NLRP11*^KO^ cells after nigericin treatment (Fig. 2e), but not after poly(dA:dT) transfection (Fig. 2f). Pyroptosis was also defective in *NLRP11*^KO^ cells comparable to *NLRP3*^KO^ cells (Fig. 2g). Detection of ASC and NLRP3 was strongly reduced in the culture SNs of primed and nigericin-activated *NLRP11*^KD^ cells compared to Ctrl^KD^ cells but was maintained in *NLRP11*^KD^ THP-1 cell lysates (Fig. 2h), suggesting ASC and NLRP3 were retained inside the cells and not released by pyroptosis. Overall, NLRP11 regulated NLRP3-dependent caspase-1 activation, GSDMD cleavage, pyroptosis and the release of inflammasome particles.

### NLRP11 is a component of the NLRP3 inflammasome

**Fig. 3:**
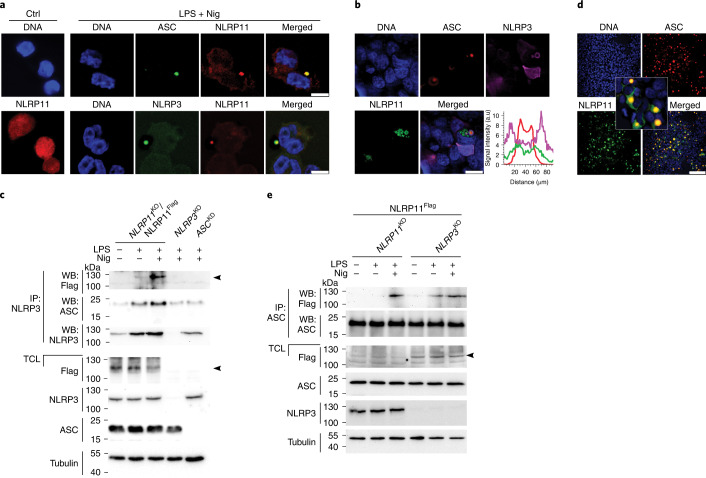
NLRP11 is an NLRP3 inflammasome component. **a**, Confocal microscopy of immunostained NLRP11, ASC, NLRP3 and DNA (DAPI) using phorbol 12-myristate-13-acetate (PMA)-differentiated THP-1 cells left untreated, primed with LPS (200 ng ml^−1^, 4 h) or primed and activated with nigericin (5 μM, 20 min). Scale bars, 10 μm. **b**, Confocal microscopy of HEK293 cells immunostained for NLRP11, ASC, NLRP3 and DNA after transient transfection with NLRP11, ASC and NLRP3; also shown is a histogram of concentric layer localization of ASC, NLRP3 and NLRP11 obtained from deconvolved 3D volumetric analysis (bottom right). Scale bar, 20 μm. **c**, Immunoprecipitation of TCLs with immobilized anti-NLRP3 antibodies of untreated, LPS-primed (200 ng ml^−1^, 2 h) and LPS-primed and nigericin (5 μM, 10 min)-treated *NLRP11*^KO^ THP-1 cells restored with NLRP11-Flag (NLRP11^Flag^) and *NLRP3*^KO^ and *ASC*^KD^ cells and immunoblot for Flag, NLRP3, ASC and tubulin loading control alongside TCLs. The weak background signal present in the ASC immunoblot, even in *AS*C^KD^ cell immunoprecipitation samples, reflects background from the antibody light chain. **c**,**e**, Arrowheads indicate the correct-size protein. **d**, Confocal microscopy of HEK293 cells immunostained for ASC, NLRP11 and DNA after transient transfection with NLRP11 and ASC. Scale bar, 100 μm. **e**, Immunoprecipitation with immobilized anti-ASC antibodies from TCLs of *NLRP11*^KO^ and *NLRP3*^KO^ cells restored with NLRP11^Flag^ left untreated, primed with LPS (200 ng ml^−1^, 2 h) and primed and activated with nigericin (5 μM, 10 min) and immunoblot of immunoprecipitates and TCLs for Flag, ASC, NLRP3 and tubulin loading control. WB, western blot. Source data

**Extended Data Fig. 5:**
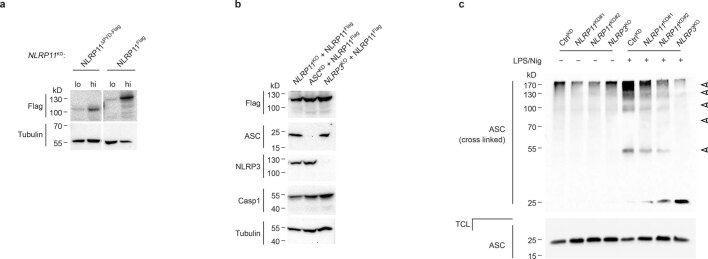
*NLRP11* silencing impairs ASC polymerization. **(a)** Immunoblot for Flag and tubulin loading control using TCL from NLRP11^Flag^ and NLRP11^ΔPYD-Flag^ cells sorted into low (lo) and high (hi) expressing populations. The gap in the blot indicates removal of additional NLRP11-expressing cell populations not included in this study. **(b)** Immunoblot for Flag, ASC, NLRP3, caspase-1 and tubulin loading control using TCL from *NLRP11*^KO^, *ASC*^KD^ and *NLRP3*^KO^ cells restored with NLRP11-Flag. **(c)** ASC crosslinking and immunoblot for ASC using TCL and crosslinked TCL from Ctrl^KD^, *NLRP11*^KD#1^, *NLRP11*^KD#2^, and *NLRP3*^KO^ cells left untreated, primed with LPS (200 ng ml^−1^, 4 h) or primed+activated with Nig (5 μM, 20 min). Arrowheads mark crosslinked ASC polymers. Source data

To determine whether NLRP11 was a part of the NLRP3 inflammasome, we stained untreated or primed and nigericin-activated cells for NLRP11 and found redistribution of diffuse NLRP11 into characteristic ‘speck’-like aggregates, which colocalized with ASC and NLRP3 upon NLRP3 activation (Fig. 3a), suggesting all three proteins formed a complex. When transiently transfected in HEK293 cells, NLRP11, ASC and NLRP3 colocalized to the characteristic ASC aggregates within concentric layers (Fig. 3b). A cross-section view indicated NLRP11 localized between the ASC core and peripheral NLRP3 (Fig. 3b), similar to the spatial organization reported for NLRP3 and NLRC4 (ref. ^28^). To directly test whether NLRP11 interacted with the NLRP3 inflammasome, we transduced NLRP11-Flag into *NLRP11*^KO^ cells (NLRP11^Flag^ cells), to allow the specific detection and selection of different levels of NLRP11 expression (Extended Data Fig. 5a). In these cells, NLRP3 copurified NLRP11^Flag^ and ASC following priming and nigericin activation, whereas NLRP3 did not bind to NLRP11^Flag^ in primed cells (Fig. 3c). To test whether NLRP11 interacted with NLRP3, ASC or both, we co-expressed NLRP11 and ASC in HEK293 cells. NLRP11 colocalized with the ASC aggregates, which are induced spontaneously after expression in HEK293 cells, in the absence of NLRP3 (Fig. 3d), suggesting that ASC could be bridging NLRP11 to the NLRP3 inflammasome. To test whether NLRP11 recruited ASC independently of NLRP3, we immunoprecipitated ASC from primed and nigericin-activated *NLRP3*^KO^ cells expressing NLRP11^Flag^ (Extended Data Fig. 5b) and observed that ASC interacted with NLRP11^Flag^ even in the absence of NLRP3 (Fig. 3e). These data suggest that NLRP11 interacted with ASC independently of NLRP3 in response to nigericin.

### ASC recruitment and polymerization requires the NLRP11^PYD^

**Fig. 4:**
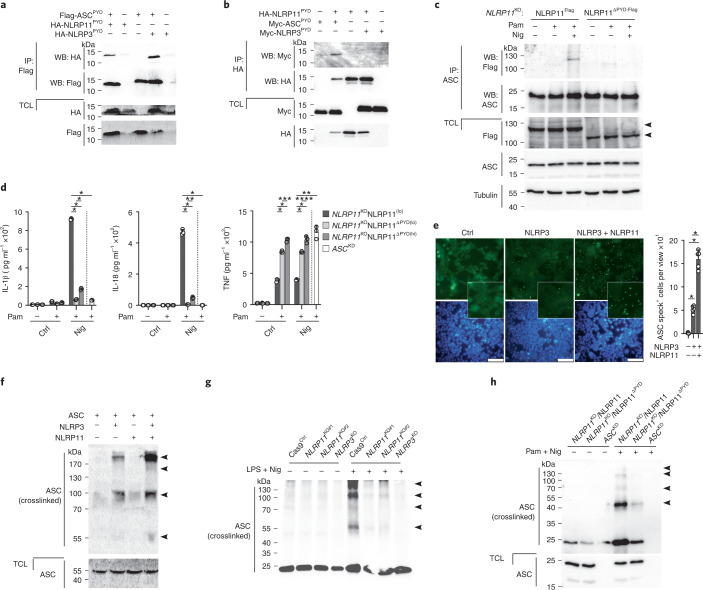
NLRP11^PYD^ recruits ASC and is necessary for efficient ASC polymerization. **a**,**b**, Immunoprecipitation with immobilized anti-Flag (a) and anti-HA (b) antibodies, and TCLs were analyzed by immunoblot for HA-, Myc- and Flag-tagged proteins after transient transfection of HEK293 cells with Flag-ASC^PYD^, HA-NLRP11^PYD^ and HA-NLRP3^PYD^ (a) and HA-NLRP11^PYD^, Myc-ASC^PYD^ and Myc-NLRP3^PYD^ (b), as indicated. **c**, Immunoprecipitation with immobilized anti-ASC antibodies from TCL of *NLRP11*^KO^ cells restored with NLRP11^Flag^ or NLRP11^ΔPYD-Flag^ left untreated, primed with Pam3CSK4 (1 μg ml^−1^, 2 h) and primed and activated with nigericin (5 μM, 10 min); immunoprecipitates and TCLs were analyzed by immunoblot for Flag, ASC and tubulin loading control. Arrowheads indicate the correct-size protein. **d**, IL-1β, IL-18 and TNF ELISA from SNs of *NLRP11*^KO^ cells restored with low-expressing NLRP11^Flag^ cells (NLRP11^lo^, NLRP11^ΔPYD(lo)^), high-expressing NLRP11^ΔPYD(hi)^ and *ASC*^KD^ cells left untreated, primed with Pam3CSK4 (1 μg ml^−1^, 4 h) and primed and activated with nigericin (5 μM, 25 min) (*n* = 3, mean ± s.d.); *P < 0.0001, **P = 0.0002, ***P = 0.0007, ****P = 0.0038. The dotted line indicates that for *ASC*^KD^, only the Pam3CSK4 + nigericin group is shown. **e**, Fluorescence microscopy of EGFP and DAPI in HEK293^ASC-EGFP^ cells transiently transfected with empty vector (Ctrl), NLRP3 and NLRP11 as indicated (left) and quantification of ASC speck^+^ cells per view (right). (*n* = 5, mean ± s.d.); *P < 0.0001, scale bars, 100 μm. **f**, immunoblot for ASC of crosslinked TCL from above cells. Arrowheads indicate oligomers. **g**,**h**, Immunoblot for ASC of TCLs and crosslinked TCLs from Cas9^Ctrl^, *NLRP11*^KO#1^, *NLRP11*^KO#2^ and *NLRP3*^KO^ cells left untreated or primed with LPS (200 ng ml^−1^, 4 h) and activated with nigericin (5 μM, 20 min) (g) and NLRP11^Flag^, NLRP11^ΔPYD-Flag^ and *ASC*^KD^ cells left untreated or primed with Pam3CSK4 (1 μg ml^−1^, 4 h) and activated with nigericin (5 μM, 15 min) (h). Arrowheads indicate oligomers. Source data

Both ASC and NLRP11 contain a PYD, which is known to mediate homotypic interactions. Indeed, the PYD of ASC (ASC^PYD^) was sufficient to copurify the NLRP11^PYD^, at levels comparable to the established ASC^PYD^–NLRP3^PYD^ interaction (Fig. 4a). However, the NLRP11^PYD^ did not copurify the NLRP3^PYD^ (Fig. 4b). To understand the role of the NLRP11^PYD^, we transduced *NLRP11*^KO^ cells with NLRP11 lacking the PYD (NLRP11^ΔPYD-Flag^), selected for comparable expression to full-length NLRP11^Flag^ (Extended Data Fig. 5a) and immunoprecipitated ASC. Although ASC coimmunoprecipitated NLRP11^Flag^, it did not copurify NLRP11^ΔPYD-Flag^ in primed and nigericin-activated cells (Fig. 4c), suggesting that the NLRP11^PYD^ was required for the NLRP11–ASC interaction. Primed and nigericin-activated NLRP11^ΔPYD-Flag^ cells also failed to secrete IL-1β and IL-18 compared to NLRP11^Flag^ cells, even at increased expression of NLRP11^ΔPYD-Flag^ (Fig. 4d), indicating that the NLRP11^PYD^ was required for NLRP3 inflammasome-mediated cytokine release. Primed and nigericin-activated NLRP11^ΔPYD-Flag^ cells also showed increased secretion of TNF compared to NLRP11^Flag^ cells, comparable to *ASC*^KD^ cells (Fig. 4d) and reminiscent of the effect observed in *NLRP11*^KO^ cells (Fig. 1h), suggesting that the NLRP11^PYD^ was also important for this non-NLRP3 inflammasome-mediated effect on NF-κB^26,27^. ASC polymerization is believed to be nucleated by activated NLRP3^10,11^. However, because NLRP11 interacted with ASC and was necessary for NLRP3 inflammasome activation, we investigated whether NLRP11 contributed to the recruitment and polymerization of ASC. In HEK293^ASC-EGFP^ cells transfected with NLRP3 at levels that promoted only limited ASC polymerization, coexpression of NLRP11 greatly enhanced ASC polymerization (Fig. 4e). Comparable results were obtained by immunoblot assays following nonreversible crosslinking of cell lysates from above cells. Expression of NLRP3 alone promoted the formation of dimeric and oligomeric ASC, whereas coexpression of NLRP3 and NLRP11 synergistically induced strong ASC polymerization (Fig. 4f). Expression of NLRP11 alone did not efficiently nucleate ASC polymerization (Fig. 4f). Primed and nigericin-activated *NLRP11*^KO^ cells (Fig. 4g) or *NLRP11*^KD^ cells (Extended Data Fig. 5c) were completely defective in ASC polymerization, similar to *NLRP3*^KO^ cells, without affecting the expression of total ASC in the cells. Only NLRP11^Flag^ cells, but not NLRP11^ΔPYD-Flag^ cells, promoted nigericin-induced ASC polymerization (Fig. 4h). These results indicated that NLRP3 can nucleate ASC polymerization when overexpressed but that both NLRP3 and NLRP11 are required to induce ASC polymerization in THP-1 cells in a manner dependent on the NLRP11^PYD^.

### NLRP11 is necessary for the oligomerization of human NLRP3

**Fig. 5:**
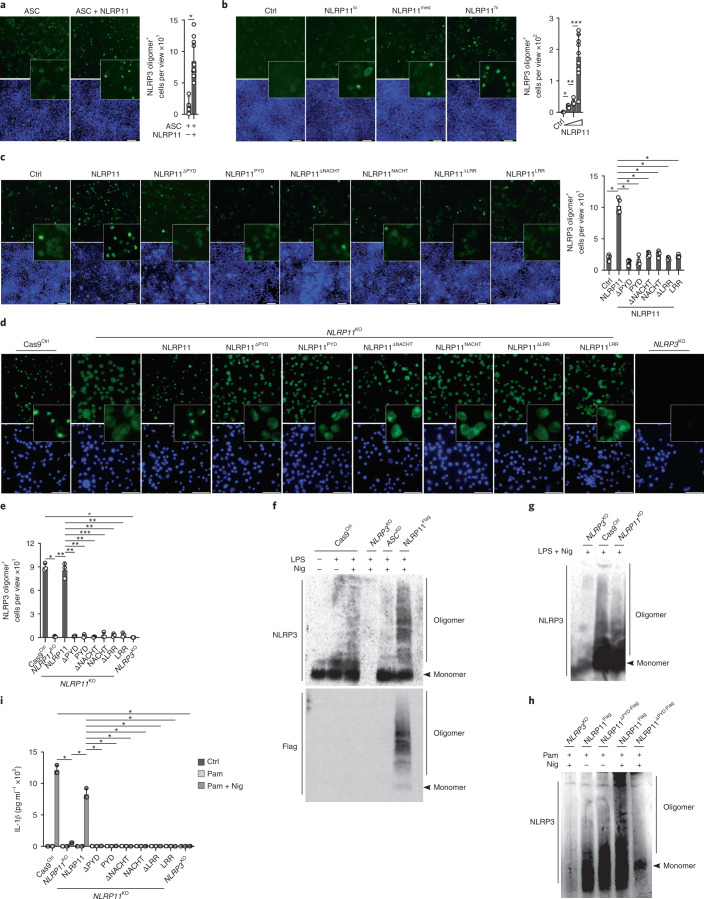
NLRP11 is necessary for oligomerization of human NLRP3. **a**–**c**, Fluorescence microscopy of EGFP and DAPI in HEK293^NLRP3-EGFP^ cells transiently cotransfected with (a) ASC and NLRP11, (b) empty plasmid (Ctrl) and increasing concentrations of NLRP11 and (c) Ctrl, NLRP11, NLRP11^ΔPYD^, NLRP11^PYD^, NLRP11^ΔNACHT^, NLRP11^NACHT^, NLRP11^ΔLRR^ or NLRP11^LRR^, as indicated (left) and presented as NLRP3 oligomer^+^ cells per view (right) (a, ASC: *n* = 4, ASC + NLRP11: *n* = 13; b, Ctrl *n* = 3, NLRP11^lo, med^: *n* = 5, NLRP11^hi^: *n* = 9; c: *n* = 5; mean ± s.d.; a, *P = 0.0003; b, *P = 0.0006, **P = 0.0164, ***P = 0.0009; c, *P < 0.0001; a–c, scale bars, 100 μm). **d**,**e**, Confocal microscopy of NLRP3 and DAPI staining, scale bars, 50 μm (d), and quantification of NLRP3 oligomer^+^ cells per view (e) using PMA-differentiated Cas9^Ctrl^, *NLRP3*^KO^, *NLRP11*^KO^ and *NLRP11*^KO^ restored with NLRP11-Flag or truncated NLRP11-Flag, as indicated primed with Pam3CSK4 (1 μg ml^−1^, 4 h) and activated with nigericin (5 μM, 25 min) (*n* = 3 mean ± s.d.); *P < 0.0001; **P = 0.0001, ***P = 0.0002. **f**–**h**, Blue native PAGE and immunoblot for NLRP3 and Flag using TCLs from Cas9^Ctrl^, *NLRP3*^KO^, ASC^KD^ and NLRP11^Flag^ cells (f), Cas9^Ctrl^, *NLRP3*^KO^ and *NLRP11*^KO^ cells (g) and NLRP11^Flag^ or NLRP11^ΔPYD-Flag^ cells (h) left untreated, primed with LPS (200 ng ml^−1^, 4 h) (f,g) or Pam3CSK4 (1 μg ml^−1^, 4 h) (h) and activated with nigericin (5 μM, 20–30 min). **i**, IL-1β ELISA using SNs from cells indicated above left untreated, primed with Pam3CSK4 (1 μg ml^−1^, 4 h) and primed and activated with nigericin (5 μM, 25 min) (*n* = 3, mean ± s.d.); *P < 0.0001. Source data

**Extended Data Fig. 6:**
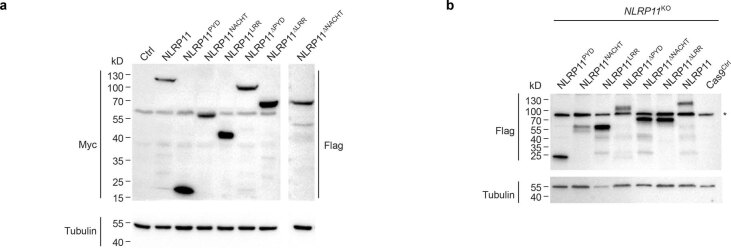
NLRP11 domain expression for NLRP3 oligomerization. **(a)** Immunoblot for Myc, Flag and tubulin loading control using TCL from HEK293^NLRP3-EGFP^ cells transiently cotransfected with empty vector (Ctrl), Myc or Flag-tagged NLRP11 or NLRP11 domains as indicated. **(b)** Immunoblot for Flag and tubulin loading control using TCL from *NLRP11*^KO^ THP-1 stably restored with NLRP11-Flag or truncated NLRP11-Flag, as indicated. *marks a cross-reactive protein. Source data

Next, we investigated whether NLRP11 directly contributed to NLRP3 oligomerization. Cotransfection of NLRP11 with ASC and NLRP3^EGFP^ in HEK293 cells enhanced NLRP3^EGFP^ oligomerization (Fig. 5a). Furthermore, NLRP11 cotransfection with dispersed NLRP3^EGFP^ in HEK293 cells promoted NLRP3 oligomerization in a dose-dependent manner, even in the absence of ASC, excluding any feedback from polymerized ASC (Fig. 5b), indicating that NLRP11 was necessary and sufficient to promote NLRP3 oligomerization. Because expression of NLRP11^ΔPYD^ in *NLRP11*^KO^ cells resulted in defective release of IL-1β and IL-18 (Fig. 4d), suggesting that the NLRP11^PYD^ may also be required for NLRP3 oligomerization, we tested the ability and requirement of individual NLRP11 domains to promote NLRP3 oligomerization. Comparable expression of NLRP11, but not NLRP11^ΔPYD^, resulted in NLRP3 oligomerization in HEK293 cells (Fig. 5c and Extended Data Fig. 6a). The PYD, NACHT or LRR alone did not support NLRP3 oligomerization (Fig. 5c). NLRP11 lacking the NACHT domain (NLRP11^ΔNACHT^) or the LRR (NLRP11^ΔLRR^) was also defective in inducing NLRP3 oligomerization (Fig. 5c), suggesting that intact NLRP11 was required. To further interrogate this mechanism, we stably restored the expression of NLRP11, NLRP11^ΔPYD^, NLRP11^ΔNACHT^, NLRP11^ΔLRR^, NLRP11^PYD^, NLRP11^NACHT^ and NLRP11^LRR^ in *NLRP11*^KO^ cells and sorted cells for comparable expression (Extended Data Fig. 6b). Primed and nigericin-activated Cas9^Ctrl^ and *NLRP11*^KO^ cells expressing NLRP11, but not *NLRP11*^KO^ cells or *NLRP11*^KO^ cells expressing any of the other truncated NLRP11 proteins, induced NLRP3 oligomerization, as determined by microscopy and quantification of NLRP3 oligomers using *NLRP3*^KO^ cells as a specificity control (Fig. 5d,e). Biochemical analysis using blue native gel electrophoresis also demonstrated the nigericin-induced NLRP3 oligomerization in primed Cas9^Ctrl^ cells, which was further enhanced in NLRP11^Flag^ cells (Fig. 5f) but reduced in *NLRP11*^KO^ cells (Fig. 5g) or NLRP11^ΔPYD-Flag^ cells (Fig. 5h). *NLRP3*^KO^ cells were used as a specificity control. This analysis also revealed the oligomerization of NLRP11 itself in primed and nigericin-activated NLRP11^Flag^ cells (Fig. 5f). Primed and nigericin-activated NLRP11^Flag^ cells, but not *NLRP11*^KO^ cells and *NLRP11*^KO^ cells expressing any truncated NLRP11, secreted IL-1β comparable to Cas9^Ctrl^ cells (Fig. 5i). Collectively, these results demonstrated that intact NLRP11 was necessary for the oligomerization of NLRP3.

### NLRP11 promotesNLRP3 inflammasome assembly

**Fig. 6:**
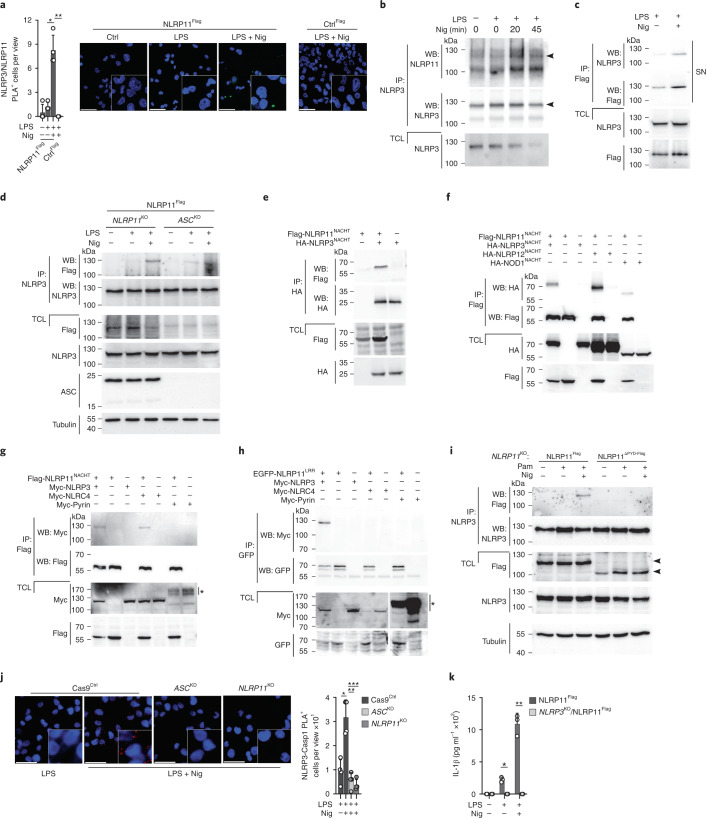
NLRP11 acts as a scaffold for NLRP3 inflammasome assembly. **a**, Confocal microscopy of PLA (green) between NLRP3 and Flag and DAPI using PMA-differentiated Ctrl^Flag^ and NLRP11^Flag^ cells left untreated, primed with LPS (200 ng ml^−1^, 4 h) and primed and activated with nigericin (5 μM, 20 min); scale bar, 50 μm; (right), and quantification of PLA^+^ cells per view (left) (*n* = 4, mean ± s.d.); *P = 0.0004; **P = 0.0001. **b**, Immunoprecipitation with immobilized anti-NLRP3 antibodies using TCLs from untreated, LPS-primed (200 ng ml^−1^, 4 h) and primed + nigericin (5 μM, 20 min and 45 min) activated THP-1 cells and immunoblot of immunoprecipitates and TCLs for NLRP11 and NLRP3. Arrowheads indicate the correct-size proteins. **c**, Immunoprecipitation with immobilized anti-Flag antibodies using SNs of NLRP11^Flag^ cells primed with LPS (200 ng ml^−1^, 4 h) and primed and activated with nigericin (5 μM, 10 min) and immunoblot of immunoprecipitates and TCLs for NLRP3 and Flag. **d**, Immunoprecipitation with immobilized anti-NLRP3 antibodies using TCLs from *NLRP11*^KO^ and *ASC*^KD^ THP-1 cells restored with NLRP11-Flag left untreated, LPS-primed (200 ng ml^−1^, 2 h) and primed and activated with nigericin (5 μM, 10 min), and immunoblot of immunoprecipitates and TCLs by immunoblot for Flag, NLRP3, ASC and tubulin loading control. **e**–**h**, Immunoprecipitation with immobilized anti-HA (e), anti-Flag (f,g) and anti-EGFP (h) antibodies using TCLs from HEK293 cells transiently transfected with Flag-NLRP11^NACHT^ and HA-NLRP3^NACHT^ (e) ; Flag-NLRP11^NACHT^, HA-NLRP3^NACHT^, HA-NLRP12^NACHT^ and HA-NOD1^NACHT^ (f); Flag-NLRP11^NACHT^, Myc-NLRP3, Myc-NLRC4 and Myc-pyrin (g); and EGFP-NLRP11^LRR^, Myc-NLRP3, Myc-NLRC4 and Myc-pyrin as indicated (h); and immunoblot of immunoprecipitates and TCLs for HA-, Flag-, Myc- and EGFP. Asterisk denotes modified pyrin (g,h). The gap in the TCLs in panel h marks an empty lane between NLRC4 and pyrin. **i**, Immunoprecipitation with immobilized anti-NLRP3 antibodies using TCLs from NLRP11^Flag^ and NLRP11^ΔPYD- Flag^ cells left untreated, primed with Pam3CSK4 (1 μg ml^−1^, 2 h) and primed and activated with nigericin (5 μM, 10 min) and immunoblot of immunoprecipitates and TCLs for Flag, NLRP3 and tubulin loading control. **j**, Confocal microscopy of PLA (red) between NLRP3 and caspase-1 and DAPI of PMA-differentiated Cas9^Ctrl^, *ASC*^KD^ and *NLRP11*^KO^ cells primed with LPS (200 ng ml^−1^, 4 h) and primed and activated with nigericin (5 μM, 20 min); scale bar, 50 μm; (left), and quantification of PLA^+^ cells per view (right) (*n* = 4, mean ± s.d.); *P = 0.0021; **P = 0.0006, ***P = 0.0003. **k**, IL-1β ELISA of SNs from Cas9^Ctrl^ and *NLRP3*^KO^ cells stably expressing NLRP11-Flag left untreated, primed with LPS (200 ng ml^−1^, 4 h) and primed and activated with nigericin (5 μM, 30 min) (*n* = 3, mean ± s.d.); *P = 0.0006, **P = 0.0003. Source data

**Extended Data Fig. 7:**
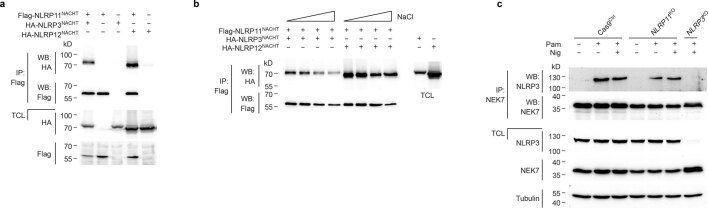
NLRP11 NACHT interactions and NLRP11 does not affect NEK7 recruitment to NLRP3. **(a,b)** Immunoprecipitation with immobilized anti-Flag antibodies using TCL from HEK293 cells transiently transfected with Flag-NLRP11^NACHT^, HA-NLRP3^NACHT^ and HA-NLRP12^NACHT^, as indicated **(a)** using RIPA buffer and **(b)** washed with increasing NaCl concentrations (200 mM, 400 mM, 600 mM and 800 mM) and immunoblot of IP and TCL for HA and Flag. **(c)** IP with immobilized anti-NEK7 antibodies using TCL from untreated, Pam3CSK4 primed (1 μg ml^−1^, 2 h) and primed+Nig (5 μM, 10 min) activated Cas9^Ctrl^, *NLRP11*^KO^ and *NLRP3*^KO^ cells and immunoblot of IP and TCL for NLRP3, NEK7 and tubulin loading control. Source data

**Extended Data Fig. 8:**
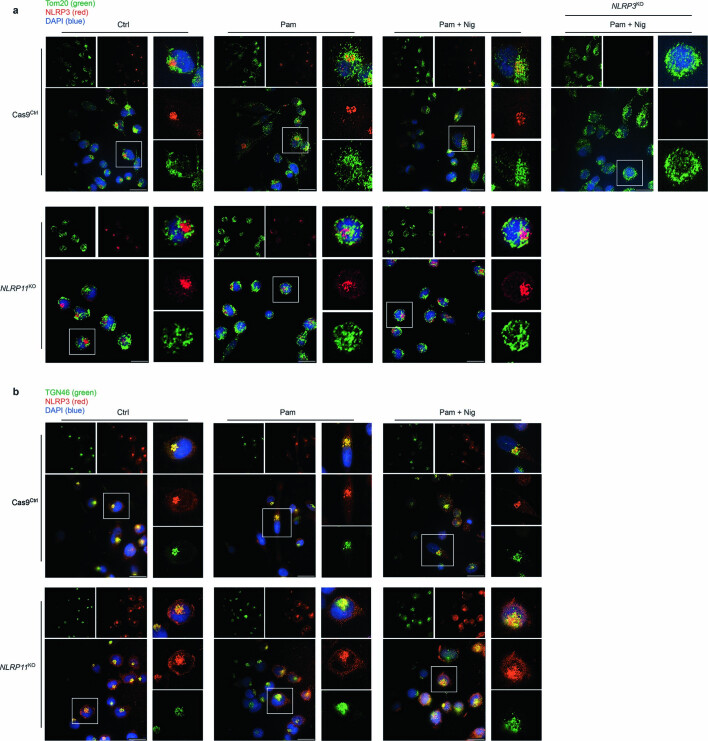
NLRP11 does not affect TGN localization of NLRP3. **(a,b)** Confocal microscopy of PMA-differentiated Cas9^Ctrl^, *NLRP11*^KO^ and *NLRP3*^KO^ cells left untreated, primed with Pam3CSK4 (1 μg ml^−1^, 2 h) or primed+activated with Nig (5 μM, 20 min) immunostained for **(a)** Tom20 (green), NLRP3 (red) and DAPI (blue), and **(b)** TGN46 (green), NLRP3 (red) an d DAPI (blue).

Next, we investigated whether NLRP11 interacted with NLRP3 using a proximity ligation assay (PLA). A specific PLA signal was detected in primed and nigericin-activated NLRP11^Flag^ cells, but not in primed cells (Fig. 6a). NLRP3 immunoprecipitation further corroborated the nigericin-dependent interaction of NLRP3 with endogenous NLRP11 in THP-1 cells (Fig. 6b). The reduced expression of NLRP3 and NLRP11 in TCL after prolonged activation (45 min) was likely the result of partially released NLRP3 inflammasome components (Fig. 6b). Accordingly, Flag immunoprecipitation from the SNs of primed and nigericin-activated, but not from primed, NLRP11^Flag^ cells copurified NLRP3 (Fig. 6c), indicating that NLRP11 and NLRP3 were released as a complex by pyroptosis. To determine whether the NLRP3–NLRP11 interaction occurred independently of ASC, we expressed NLRP11^Flag^ in *ASC*^KD^ cells (Extended Data Fig. 5b) and immunoprecipitated NLRP3. NLRP3 coimmunoprecipitated NLRP11 in primed and nigericin-activated NLRP11^Flag^ cells, and this interaction was also observed in *ASC*^KD^ cells expressing NLRP11^Flag^ (Fig. 6d), suggesting that NLRP11 interacted with NLRP3 independently of ASC. NLRP3 oligomerization is mediated by the NACHT domain^8^. In HEK293 cells, the NLRP11^NACHT^ bound directly to the NLRP3^NACHT^, as demonstrated by coimmunoprecipitation of transiently transfected NLRP11^NACHT^ and NLRP3^NACHT^ domains (Fig. 6e), but the NLRP11^NACHT^ also bound to the NLRP12^NACHT^ and NOD1^NACHT^ domains (Fig. 6f). Binding was still observed under very stringent conditions in RIPA buffer (Extended Data Fig. 7a) and in buffers with up to 800 mM NaCl (Extended Data Fig. 7b), indicating that the NLRP11^NACHT^ bound with high affinity to other NACHT domains. To address whether isolated NACHT domains were more easily accessible for interactions in the absence of intramolecular interactions with the LRR and/or the PYD in the intact protein^9,29^, we tested the interaction of the NLRP11^NACHT^ with full-length NLRP3 and NLRC4 by transient transfection of HEK293 cells. The NLRP11^NACHT^ coimmunoprecipitated NLRP3 and NLRC4 (Fig. 6g), even though the NLRC4 inflammasome was not affected by NLRP11 (Fig. 1j), confirming previous reports of spontaneous interactions between NLR NACHT domains in HEK293 cells^30^. The NLRP11^NACHT^ did not interact with pyrin, which lacks a NACHT domain (Fig. 6g). These results suggested that although the NLRP11^NACHT^ can interact with the NLRP3^NACHT^, additional events may facilitate and determine the specificity of these interactions in macrophages. NEK7 promotes NLRP3 oligomerization and ASC polymerization but cannot mediate NLRP3 activation on its own^20–22^. NEK7 binds to the LRR and NACHT domains of NLRP3 to bridge two adjacent NLRP3 molecules^23^. NEK7 interacted with NLRP3 in LPS-primed and LPS-primed and nigericin-activated Cas9^Ctrl^ and *NLRP11*^KO^ cells (Extended Data Fig. 7c), indicating that NLRP11 does not mediate the NEK7–NLRP3 interaction. We did not observe substantial NLRP3 localization to the mitochondria in untreated, primed and primed and nigericin-activated Cas9^Ctrl^ and *NLRP11*^KO^ cells (Extended Data Fig. 8a), but, as previously described^31^, NLRP3-activating stimuli caused the disassembly of the trans-Golgi network (TGN), and NLRP3 localization to the dispersed TGN in Cas9^Ctrl^ cells and also in *NLRP11*^KO^ cells (Extended Data Fig. 8b), indicating that NLRP11 did not affect the intracellular localization of NLRP3. Because the LRR has a key role in assembling mouse NLRP3 oligomers^32^, we tested whether the NLRP11^LRR^ was involved in the NLRP3–NLRP11 interaction. Transient transfection of HEK293 cells with NLRP11^LRR^ and NLRP3, NLRC4 (which has a LRR) or pyrin (which lacks a LRR) demonstrated that the NLRP11^LRR^ coprecipitated NLRP3, but not NLRC4 or pyrin (Fig. 6h). NLRP3 coimmunoprecipitated NLRP11 in primed and nigericin-activated NLRP11^Flag^ cells, but not in NLRP11^ΔPYD-Flag^ cells (Fig. 6i). To test whether NLRP11 directly controlled the assembly of the NLRP3 inflammasome, we performed a PLA between NLRP3 and caspase-1. A positive PLA signal was detected in primed and nigericin-activated Cas9^Ctrl^ cells, but not in *NLRP11*^KO^ or *ASC*^KD^ cells (Fig. 6j). However, expression of NLRP11 cannot compensate for the loss of NLRP3, as NLRP11 overexpression could not induce IL-1β release in primed and nigericin-activated *NLRP3*^KO^ cells (Fig. 6k). Collectively, these results showed that NLRP11 interacts with NLRP3 independently of ASC through its NACHT and LRRs, but an intact NLRP11, including the NLRP11^PYD^, was nevertheless required for promoting nigericin-induced interactions between NLRP11 and NLRP3.

### NLRP11 is necessary for IL-1β release in CAPS

**Fig. 7:**
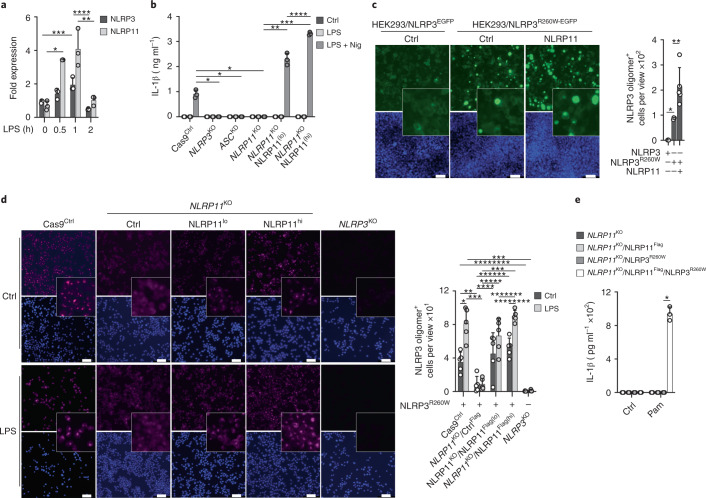
NLRP11 is necessary for IL-1β release in CAPS. **a**, qPCR of *NLRP3* and *NLRP11* mRNA levels from THP-1 cells primed with LPS (200 ng ml^−1^) as indicated, presented as fold expression to average mRNA levels present in uninduced cells (*n* = 3, mean ± s.d.); *P < 0.0001, **P = 0.0103, ***P = 0.0145, ****P = 0.0037. **b**, IL-1β ELISA of SN from Cas9^Ctrl^, *NLRP11*^KO^, *NLRP3*^KO^, *ASC*^KD^, NLRP11^Flag(lo)^ and NLRP11^Flag(hi)^ cells primed with LPS (200 ng ml^−1^, 4 h) and primed and activated with nigericin (5 μM, 30 min) (*n* = 3, mean ± s.d.); *P = 0.0006; **P = 0.0012, ***P < 0.0001, ****P = 0.0021. **c**, Fluorescence microscopy of EGFP and DAPI in HEK293^NLRP3-EGFP^ and HEK293^NLRP3R260W-EGFP^ cells transiently cotransfected with empty vector (Ctrl) or NLRP11; scale bars, 100 μm (left), and quantification of NLRP3 oligomer^+^ cells per view (right) (Ctrl: *n* = 3, NLRP11: *n* = 5, mean ± s.d.); *P < 0.0001; **P = 0.029. **d**, Confocal microscopy of DAPI and NLRP3 immunostained PMA-differentiated Cas9^Ctrl^, *NLRP11*^KO^, NLRP11^Flag(lo)^ and NLRP11^Flag(lo)^ and *NLRP3*^KO^ cells stably expressing NLRP3^R260W^ left untreated (Ctrl) or primed with LPS (200 ng ml^−1^, 4 h); scale bars, 50 μm (left), and quantification of NLRP3 oligomer^+^ cells per view (right) (*n* = 5, mean ± s.d.); *P = 0.0017; **P = 0.0046, ***P < 0.0001, ****P = 0.0153, *****P = 0.0003, ******P = 0.0001, *******P = 0.0427, ********P = 0.0002. **e**, IL-1β ELISA of SNs from *NLRP11*^KO^ and NLRP11^Flag^ cells stably expressing NLRP3^R260W^ left untreated (Ctrl) or primed with Pam3CSK4 (1 μg ml^−1^, 2 h) (*n* = 3, mean ± s.d.). *P < 0.0001. Source data

**Extended Data Fig. 9:**
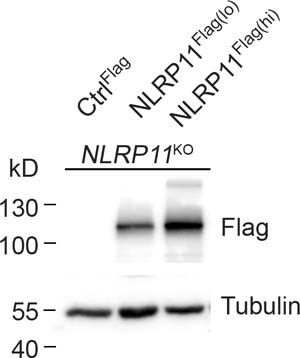
Low and high-expressing NLRP11^Flag^ cells. Immunoblot for Flag and tubulin loading control using TCL of Ctrl^Flag^, NLRP11^Flag(lo)^ and NLRP11^Flag(hi)^ cells. Source data

qPCR analysis indicated that *NLRP11* mRNA was induced in primed THP-1 cells, similar to the inducible expression of *NLRP3* (Fig. 7a). In primed *NLRP11*^KO^ cells restored with low or high amounts of NLRP11 protein to mimic the inducible expression of NLRP11-primed cells (Fig. 7b), we observed an NLRP11 concentration-dependent increase in IL-1β secretion in response to nigericin treatment (Fig. 7c), indicating that the activation of the NLRP3 inflammasome was influenced by the amount of NLRP11. In patients with CAPS, myeloid-lineage restricted mutations in NLRP3 and somatic mosaicism^6^ allow NLRP3 activation in the absence of an activation signal, and priming alone is sufficient to trigger NLRP3 inflammasome-mediated IL-1β release^33^. The majority of CAPS mutations are localized within the NACHT domain and prevent the autoinhibited conformation of NLRP3 (ref. ^34^). Accordingly, stable expression of the CAPS mutation NLRP3^R260W-EGFP^ in HEK293 cells resulted in spontaneous oligomerization of NLRP3 (Fig. 7d), whereas wild-type NLRP3^EGFP^ was distributed diffusely throughout the cells (Fig. 7d). Coexpression of NLRP11 in HEK293 cells further increased the aggregation of NLRP3^R260W-EGFP^ (Fig. 7d), indicating that NLRP11 even enhanced the oligomerization of the constitutively active NLRP3^R260W^. Stable expression of NLRP3^R260W^ also resulted in spontaneous oligomerization of NLRP3^R260W^ in Cas9^Ctrl^ cells when immunostained for NLRP3 (Fig. 7e), but not in *NLRP11*^KO^ cells (Fig. 7e), whereas stable reexpression of NLRP11^Flag^ in *NLRP11*^KO^ cells restored NLRP3^R260W^ oligomerization in a dose-dependent manner (Fig. 7e). Priming further increased the number of NLRP3^R260W^ oligomers in Cas9^Ctrl^ cells, but not in *NLRP11*^KO^ cells (Fig. 7e). IL-1β was already released in primed NLRP11^Flag^ cells, but not in *NLRP11*^KO^ cells expressing NLRP3^R260W^ (Fig. 7f). These results indicated that NLRP11 was also necessary for facilitating oligomerization and promoting IL-1β release from mutant NLRP3 that causes CAPS. Therefore, NLRP11 is an essential component of the NLRP3 inflammasome (Extended Data Fig. 9).

## Discussion

Here, we identified NLRP11 as an essential component of the NLRP3 inflammasome in human macrophages, which was required for caspase-1 activation, release of IL-1β and IL-18 and pyroptosis. NLRP11 bound to ASC through homotypic PYD–PYD interactions, which was required for nigericin-induced ASC polymerization. NLRP11 also independently interacted with NLRP3, which involved the NLRP11^LRR^ and the NLRP11^NACHT^ domains and facilitated NLRP3 oligomerization.

The NLRP11^NACHT^ did not specifically interact with NLRP3 in HEK293 cells. This unspecific affinity of NACHT domains when expressed in HEK293 cells has been reported earlier^30^. In THP-1 cells, the interaction between NLRP11 and NLRP3 occurred after NLRP3 activation. Therefore, we speculate that in macrophages additional unknown signals may be required to confer specificity to NLRP11^NACHT^ domain interactions. Additional specificity is provided by the NLRP11^LRR^, which interacted with NLRP3, but not with NLRC4. Taken together, it is very likely that the NLRP11^LRR^ and the NLRP11^NACHT^ domains both contributed to the specific interaction between NLRP3 and NLRP11, reminiscent of the interaction between NLRP3 and NEK7 (ref. ^23^). Other NACHT domain-mediated NLR hetero-oligomerizations have been described, including NLRC4-NLRP3 (refs. ^28,35^), NAIP-NLRC4 (refs. ^36,37^) and Nod2-NLRP1 (ref. ^38^). NLRC5 also interacts with the NLRP3^NACHT^ to regulate NLRP3 by an unknown mechanism^39^ but has more recently been linked to major histocompatibility complex class I transactivation^40,41^. Even though NLRP11 and NLRP3 did not interact through their PYDs, the NLRP11^PYD^ was still crucial for complex formation, because deletion of the PYD prevented NLRP11 recruitment to the NLRP3 inflammasome, NLRP3 oligomerization and NLRP3 inflammasome responses, which required an intact NLRP11 protein. NLRP3 can nucleate ASC polymerization *in vitro*, and we observed this ability in HEK293 cells, but only if NLRP3 was overexpressed. Increasing the expression of NLRP3 during inflammasome priming contributes to, but is not sufficient for, inflammasome activation^3–5^. Low-level expression of NLRP3 did not nucleate ASC polymerization in HEK293 cells, and even priming-induced elevation of NLRP3 expression was insufficient in THP-1 cells in the absence of NLRP11. NLRP11 was required for NLRP3 inflammasome responses in a dose-dependent manner, but NLRP11 expression was not able to compensate for the loss of NLRP3, indicating that NLRP11 alone could not assemble an inflammasome under these conditions. This mode of activation is unique, because NLRP11 interacted with NLRP3 as well as ASC, and all three were required for NLRP3 inflammasome assembly in THP-1 cells. Other described mechanisms for inflammasome activation require bridging of the NLRP3–ASC interaction by GBP5^24^, or bridging NLRP3 molecules through NACHT-LRR interaction by NEK7 (ref. ^23^). NLRP11 uniquely combines these mechanisms. NLRP11 was required for the response to all tested soluble and crystalline NLRP3 triggers, supporting its essential role within the NLRP3 inflammasome.

NLRP11 is encoded in humans and absent from mice^42^, but whether this mechanism is unique to human macrophages will require additional studies. Nevertheless, several other examples exist for increased complexity of inflammasome regulation in humans, including the family of PYD- and CARD-only proteins^43^. In addition to its function in NLRP3 inflammasome activation, NLRP11 could potentially function as an inflammasome sensor. Arguably, this would require the ability of NLRP11 to nucleate ASC polymerization, and based on our ASC^EGFP^ polymerization assays in HEK293 cells, some NLRP11-mediated ASC polymerization was possible, especially in cells with sufficiently high NLRP11 expression. However, expression of NLRP11 in THP-1 cells failed to polymerize ASC in the absence of activated NLRP3, suggesting that physiological amounts of NLRP3 require the cooperation between NLRP3 and NLRP11, even though macrophages are the cells with the highest expression of NLRP3 (ref. ^44^).

Little is known about NLRP11, and there are conflicting reports on its role in type I interferon (IFN) or NF-κB signaling^26,27,45^. NLRP11 causes degradation of TRAF6 to inhibit TLR-mediated NF-κB activation^27^, and we observed slightly elevated TNF release in *NLRP11*^KO^ cells. However, NF-κB-dependent IL-6 release and *IL1B* transcription were not impacted. NLRP11 also binds to DDX3X and inhibits IFN-β and reduces caspase-1 activity in HEK293T cells^46^. siRNA-mediated silencing of NLRP11 in THP-1 cells slightly elevates Sendai virus-induced IFN-β production and does not affect IL-1β release^45^, but Sendai virus already completely prevents NLRP3 inflammasome assembly^47^. Several other NLRs, including NLRP2, NLRP3, NLRP6, NLRP7, NLRP12 and NLRC5, have been linked to inflammasomes, as well as transcriptional responses through regulating NF-κB, mitogen-activated protein kinase and IFN signaling^48^. Overall, our identification of NLRP11 as an essential adaptor or scaffold for NLRP3 inflammasome assembly and activation provides important insights into the still incompletely understood NLRP3 inflammasome response in humans. NLRP3 is uniquely positioned as a central sensor for infections and cellular stress and has been implicated in a wide range of inflammatory diseases ranging from crystal arthropathies to hereditary autoinflammatory disorders^49^. NLRP11 may provide an important checkpoint control for NLRP3 inflammasome assembly. Intriguingly, NLRP11 is also necessary for NLRP3 inflammasome responses initiated by CAPS-linked NLRP3 mutations, which may have important clinical implications.

## Methods

### Reagents and antibodies

The following antibodies were used: custom-raised NLRP11 rabbit polyclonal (AAMRTSNTASRQPL) and mouse monoclonal (recombinant, amino acids 105–624 and WSLKEGREIGVTPA), NLRP11 rabbit polyclonal antibodies (ab105408, Abcam; HPA046402, Millipore-Sigma and NBP1-92186, Novus Biologicals), NLRP3 mouse monoclonal (Cryo-2, Adipogen) and rabbit monoclonal (D4D8T, Cell Signaling Technology), ASC rabbit polyclonal (N-15, Santa Cruz Biotechnology), ASC rabbit polyclonal and mouse monoclonal^13,50^, caspase-1 rabbit monoclonal (D7F10, Cell Signaling Technology), cleaved caspase-1 rabbit monoclonal (D57A2, Cell Signaling Technology), GSDMD rabbit monoclonal (L60, Cell Signaling Technology), cleaved GSDMD rabbit monoclonal (E7H9G, Cell Signaling Technology), caspase-4 rabbit polyclonal (4450, Cell Signaling Technology), NEK7 rabbit monoclonal (EPR4900, Abcam), TGN46 rabbit monoclonal (JF1-024, Invitrogen, rabbit polyclonal Tom20 (FL-145, Santa Cruz Biotechnology), c-myc mouse monoclonal (9E10, Santa Cruz Biotechnology and 9B11, Cell Signaling Technology), HA mouse monoclonal (F-7, Santa Cruz Biotechnology), mouse monoclonal Flag (M2, Millipore-Sigma), mouse monoclonal tubulin (AA12.1, DSHB), rabbit polyclonal vinculin (AB6039, Millipore-Sigma) antibodies, mouse monoclonal Flag agarose (M2, Millipore-Sigma), mouse monoclonal HA agarose (HA-7, Millipore-Sigma) and HRP-conjugated anti-mouse, anti-rabbit and anti-goat IgG (H + L) (Invitrogen), and goat anti-rabbit and anti-mouse Alexa Fluor 488-, 546- and 647-conjugated antibodies (Invitrogen). Antibodies were also used as directly HRP-conjugates for western blot detection of coimmunoprecipitation experiments.

### Cell culture

THP-1 cells (TIB-202, ATCC) were maintained in RPMI 1640 media, supplemented with 10% FBS, 1 mM HEPES buffer, 2 mM glutamine, 1 mM sodium pyruvate, 100 IU ml^−1^ penicillin, 1 mg ml^−1^ streptomycin and 0.05 mM 2-mercaptoethanol; used at low passage numbers; and screened routinely for mycoplasma infections (MycoAlert, Lonza). Blood from healthy donors was drawn by the Cedars Sinai Blood Bank after obtaining informed consent under a protocol approved by Cedars Sinai Institutional Review Board and deidentified. Human peripheral blood mononuclear cells were isolated by Ficoll-Hypaque centrifugation (Millipore-Sigma) from healthy donor buffy coats and countercurrent centrifugal elutriation in the presence of 10 μg ml^−1^ polymyxin B using a JE-6B rotor (Beckman Coulter), as described earlier^51^. To ensure the purity of peripheral blood mononuclear cells, cells were washed in Hank’s buffered salt solution and resuspended in serum-free RPMI for 1 h, followed by culturing in complete medium supplemented with 20% FBS for 7 days to differentiate peripheral blood macrophages, which were then cultured in medium supplemented with 10% FBS. Isolated and differentiated peripheral blood macrophages were routinely phenotyped to ensure >85% purity, as determined by flow cytometry for CD45 and CD14. HEK293 cells (CRL-3216, ATCC) and Lenti-X HEK293 cells (632180, Takara Bio) were maintained in DMEM containing 10% FBS, 100 IU ml^−1^ penicillin and 1 mg ml^−1^ streptomycin. THP-1 cells or primary human macrophages were primed with ultrapure LPS (0111:B4, 200 ng ml^−1^, Invivogen, 4 h) or Pam3CSK4 (1 μg ml^−1^, Invivogen, 4 h). Where indicated, cells were also treated with nigericin (5 μM, Invivogen, 10–45 min), CTB (20 μg ml^−1^, List Biological Laboratories, 6 h), silica (200 μg ml^−1^, Invivogen, 6 h), TcdB (10 μg ml^−1^, R&D Systems, 8 h) and ATP (5 mM, Millipore-Sigma, 25 min); cultured in K^+-^free medium (0.8 mM MgCl_2_, 1.5 mM CaCl_2_, 10 mM HEPES, 5 mM glucose and 140 mM NaCl, pH 7.2, 3 h)^50^; or transfected with flagellin (500 ng ml^−1^, Invivogen, 4 h), poly(dA:dT) (1 μg ml^−1^, Invivogen, 4 h) or FSL-1 (0.2 μg ml^−1^, Invivogen, 4 h) and ultrapure LPS (1 μg ml^−1^, 4 h) with Lipofectamine 2000 (Invitrogen) or as otherwise indicated.

### Gene expression, silencing and knockout

NLRP11 cDNA was amplified by PCR from a human cDNA library cloned into custom pcDNA3 or pLEX expression vectors with Myc, Flag or EGFP tags. NLRP11-TAP was cloned into pHIV-IRES-dTomato (a gift from B. Welm, Addgene, plasmid 21374). Myc-tagged NLRP3^R260W^ was generated previously^13^ and subcloned into pCIG3 (pCMV-IRES-GFPv3; a gift from F. Goodrum, Addgene, plasmid 78264)^52^. NLRP11^ΔPYD^ (aa 104–1,033), NLRP11^ΔNACHT^ (Δ aa 104–560), NLRP11^ΔLRR^ (aa 1–560), NLRP11^PYD^ (aa 1–91), NLRP11^NACHT^ (aa 105–624), NLRP11^LRR^ (aa 560–1,033), NLRP3^PYD^ (aa 1–89), NLRP3^NACHT^ (aa 220–389), NOD1^NACHT^ (aa 133–435), NLRP12^NACHT^ (aa 212–528) and NLRC4 were synthesized (IDT, Genewiz) or generated by PCR, cloned into modified pcDNA3 and pHIV-IRES-dTomato expression plasmids. pcDNA3-ASC^PYD^ (aa 1–91), NLRP3 and pyrin have been described earlier^51,53,54^. All expression constructs were sequence verified. To express or restore NLRP11, NLRP11^ΔPYD^, NLRP11^ΔNACHT^, NLRP11^ΔLRR^, NLRP11^PYD^, NLRP11^NACHT^, NLRP11^LRR^ or NLRP3^R260W^ in THP-1 cells, recombinant lentivirus was produced in Lenti-X HEK293 cells by Xfect (Takara Bio) or Lipofectamine 2000 (Invitrogen)-based transfection with modified pLEX (Open Biosystems), pHIV-IRES-dTomato or pCIG3 expression plasmids encoding NLRP3^R260W^, Myc-NLRP11, NLRP11-Flag, NLRP11^ΔPYD^-Flag, NLRP11^ΔNACHT^-Flag, NLRP11^ΔLRR^-Flag, NLRP11^PYD^-Flag, NLRP11^NACHT^-Flag or NLRP11^LRR^-Flag using empty Myc and Flag vector controls and the viral packaging plasmids psPAX2 (a gift from D. Trono, Addgene, plasmid 12259) and psPAX2 (a gift from D. Trono, Addgene, plasmid 12260), followed by 0.45 μm filtration of virus-containing culture SNs. THP-1 cells were transduced with lentiviral particles in the presence of polybrene (0.45 μg ml^−1^) and MISSION ExpressMag magnetic beads (Millipore-Sigma). Cells were puromycin selected (1 μg ml^−1^) 48 h after infection for 2 weeks and sorted by flow cytometry for comparable NLRP3^R260W^, NLRP11 or truncated NLRP11 expression. NLRP11- and NLRP11^ΔPYD-^expressing THP-1 cells were further sorted into low- and high-expressing cell populations and expression verified and normalized by immunoblot.

siRNA-mediated silencing of *NLRP11* was achieved by electroporation or transfection of pooled *NLRP11* siRNA#1 (sc-61142, Santa Cruz Biotechnology) and siRNA#2 (caacauaagauucgaguua; Thermo Scientific) and non-targeting control siRNAs (Santa Cruz Biotechnology and Thermo Scientific). The siRNA for *NLRP3* has been described^51^. 1.5 ×10^6^ THP-1 cells were electroporated with single or pooled siRNA duplexes (120 nM) using the Neon Transfection System (Invitrogen) (voltage, 1,600 V; width, 10 ms; three pulses). Primary human macrophages were transfected using F2/virofect (Targeting Systems) and analyzed 72 h after transfection, as described earlier^50,51,55,56^.

shRNA-mediated *NLRP11*^KD^ was achieved via recombinant lentiviral particles produced as described above. Four distinct shRNAs were cloned into pLKO.1 vector (Addgene, plasmid 10878)^57^. shRNA#1: 5′-ccggcaacactcataaagaccgttactcgagtaacggtctttatgagtgttgttttttg-3′; shRNA#2: 5′-ccggccaactgcatgttggtgaatactcgagtattcaccaacatgcagttggttttttg-3′; shRNA#3: 5′-ccggccgttacaagttcatacacttctcgagaagtgtatgaacttgtaacggttttttg-3′; shRNA#4: 5′-ccgggatgcaagagaattaggactactcgagtagtcctaattctcttgcatcttttttg-3′ (Sigma: TRCN0000128196, TRCN0000149602, TRCN0000146572, TRCN0000128281, respectively). shNLRP11#1 was produced using shRNA#1 and shNLRP11#2 was produced from a virus pool containing all four shRNAs.

*NLRP11*^KO^ THP-1 cells were generated by CRISPR/Cas9 targeting. Four gRNAs were designed with E-CRISP and CHOPCHOP: gRNA#1: 5′-gagaagcaagatggcagaat-3′; gRNA#2: 5′-cgtgttgccaatctcttatg-3′; gRNA#3: 5′-gtgttgccaatctcttatga-3′; gRNA#4: 5′-tgcgtaaggaagatctttgtagg-3′ using lentiCRISPRv1 (Addgene, plasmid 49535)^58^ and control lentiCRISPRv1 (Cas9^Ctrl^). Cells were puromycin selected (1 μg ml^−1^) and individual cells were sequence analyzed following PCR amplification of the targeting sequence (forward: 5′-tgccaagatcagtcgacaag-3′; reverse: 5′-ggaagtgtgagagggaggtg-3). To eliminate potential expression of any alternative spliced NLRP11, we sequentially targeted the NACHT/NAD by transient electroporation of gRNAs cloned into pSpCas9(BB)-2A-GFP (Addgene, plasmid 48138) or empty vector^59^ with 4–7 μg endotoxin-free plasmid as described above. gRNA#1: 5′-ggagaaaattcatgctgcaa-3′; gRNA#2: 5′-gcagctgtcgaatgggaaga-3′; gRNA#3: 5′-gctcggcaaaagaatattcg-3′; gRNA#4: 5′-cgatggtagacagcttcaag-3′. Cells were FACS selected and individual cells were sequence analyzed following PCR amplification of the targeting sequence (forward 1: 5′-aatcgcttgaacctgggagg-3′; reverse 1: 5′-agaaacagtcctcctgcagc-3; forward 2: 5′-ccgagtcgccatcttatgct-3′; reverse 2: 5′-aagttcttcatggccccgag-3). gRNA#4 resulted in a premature stop within the NACHT/NAD. *CASP4*^KO^ THP-1 cells were generated by CRISPR/Cas9 using lentiCRISPRv1 (Addgene, plasmid 49535)^58^. *CASP4* gRNA: 5′-tggtgttttggataacttgg-3′ and Ctrl gRNA: 5′-acggaggctaagcgtcgcaa-3′. *NLRP3*^KO^, *CASP1*^KO^, and *ASC*^KD^ cells were described earlier^13,25^.

### Immunoprecipitation

HEK293 cells were transiently transfected with NACHT or PYD constructs in 12-well plates (Lipofectamine 2000, Invitrogen) and analyzed 36 h post transfection. For coimmunoprecipitation of transiently transfected HEK293 cells, cells were washed and lysed in 50 mM HEPES, pH 7.4, 10% Glycerol, 2 mM EDTA, 1% NP-40, supplemented with protease inhibitors using 100 mM NaCl (PYRIN domain), 150 mM NaCl (LRR), 180–800 mM NaCl (NACHT domain), or lysed in RIPA buffer (10 mM Tris-HCl, pH 8.0, 1 mM EDTA, 0.5 mM EGTA, 1% Triton X-100, 0.1% sodium deoxycholate, 0.1% SDS and 140 mM NaCl) supplemented with protease inhibitors (NACHT domain). THP-1 cells were treated as indicated, washed and lysed in 50 mM HEPES, pH 7.4, 50–150 mM NaCl, 10% glycerol, 2 mM EDTA, 0.5% Triton X-100, supplemented with protease inhibitors. For coimmunoprecipitation of culture SNs, conditioned media were adjusted to 0.1% Triton X-100 and supplemented with protease inhibitors and washed with 50 mM NaCl and 0.1% Triton X-100. For IP of endogenous NLRP11, cells were lysed in RIPA buffer (10 mM Tris-HCl, pH 8.0, 1 mM EDTA, 0.5 mM EGTA, 1% Triton X-100, 0.1% sodium deoxycholate, 0.1% SDS and 140 mM NaCl), supplemented with protease and phosphatase inhibitors. Cleared lysates were subjected to IP by incubating with immobilized antibodies or primary antibodies and agarose A/G beads (Santa Cruz Biotechnology) as indicated for 4–16 h at 4 °C. Following extensive washing with lysis buffer, bound proteins in Laemmli sample buffer were separated by SDS/PAGE, transferred to polyvinylidene fluoride membranes, blocked (5% non-fat dry milk, 0.1 M Tris-buffered saline, pH 7.4, 0.1 % Tween 20) and analyzed by immunoblotting as indicated using HRP-conjugated primary or secondary antibodies, ECL detection (SuperSignal West Femto, Thermo Scientific), and digital image acquisition (Thermo iBright and Ultralum Omega 14vR). TCL (5%) were also analyzed where indicated. To re-use membranes, bound antibodies were stripped (0.1 M Glycine, 2% SDS) and washed (0.1 M Tris-buffered saline, pH 7.4, 0.1 % Tween 20).

### Immunoblot

TCLs were directly collected in Laemmli buffer and serum-free culture SNs were collected, adjusted to 5% (v/v) ice-cold Trichloroacetic acid (TCA), incubated on ice for 10 min and centrifuged at 21,000×*g* for 5 min. Pellets were washed twice with ice-cold acetone, briefly air-dried and resuspended in Laemmli buffer, sonicated and analyzed by SDS/PAGE/immunoblot as described above.

### Immunofluorescence analysis

THP-1 cells were differentiated using PMA (20 nM, 16 h) on coverslips in 12-well plates (0.5 × 10^6^ cells per well), washed in PBS and rested for 48 h before treatment. HEK293 cells were transiently transfected and then seeded onto coverslips. Cells were fixed (3.7% paraformaldehyde in PBS, 20 min, room temperature), permeabilized (0.5% Triton X-100 in PBS, 10 min, room temperature), blocked (2% BSA, 2% (v/v) goat sera, 0.5% Triton X-100 in PBS, 1 h, room temperature), immunostained overnight with primary antibodies in a humidified chamber, washed and subsequently incubated with Alexa Fluor-conjugated secondary antibodies for 1 h, washed and mounted onto slides with Prolong glass antifade mountant with Nucblue stain (Invitrogen). Images were captured on a Nikon TE2000E2-PFS with ×60 and ×100 oil objectives) with image deconvolution (NIS Elements) and a Zeiss LSM 780.

### qPCR

Total RNA was isolated from cells using the E.Z.N.A. total RNA isolation Kit (Omega Bio-tek), incubated with DNAse I and reverse transcribed (Verso cDNA Synthesis Kit, Thermo Scientific). Multiplexed gene expression analysis was performed on an ABI 7300 Real-Time PCR Machine (Applied Biosystems) and Quantstudio 3 (Thermo Scientific) and displayed as relative expression compared to *ACTB*, using FAM-labeled exon-spanning primers for *IL1B* (Hs01555410_m1), *NLRP11* (Hs00935472_m1), and *NLRP3* (Hs00918082_m1) in combination with VIC-labeled primers for *ACTB* (Hs99999903_m1) (Invitrogen).

### ELISA

Cells were seeded into six-well plates (10^6^ cells per well) and treated as indicated, and cleared culture SNs were analyzed for IL-1β (Invitrogen), IL-18 (R&D Systems), IL-6 (BD Biosciences) or TNF (Invitrogen) secretion by ELISA according to the manufacturer’s instructions.

### LDH cytotoxicity assay

LDH activity was determined using the LDH Cytotoxicity Detection Kit (Takara Bio) in freshly collected culture SNs. Cytotoxicity was defined as a percentage of released LDH compared to total LDH activity upon cell lysis with 1% Triton X-100.

### Caspase-1 activity by FLICA

Primed THP-1 cells were treated with nigericin (5 μM, Invivogen, 45 min) or transfected with poly(dA:dT) (6 ng ml^−1^, Invivogen, 4 h) and simultaneously incubated with a cell-permeable, biotin labeled irreversible caspase-1 inhibitor substrate (YVAD-CMK, 20 μM) (AnaSpec). Cells were washed twice with cold PBS, fixed with 2% paraformaldehyde (Electron Microscopy Sciences) for 20 min, washed twice with PBS, permeabilized with Cytofix/Cytoperm (BD Biosciences) for 20 min at 4 °C, washed twice with Perm/Wash buffer (BD Biosciences), stained with Alexa Fluor 647-conjuagted Streptavidin (Invitrogen) and washed twice with Perm/Wash buffer. Cells were then washed twice with cold autoMACS Running Buffer (Miltenyi Biotec), resuspended in autoMACS Running Buffer and analyzed on an LSRII (BD Biosciences) and Northern Lights (Cytek) instruments. Data were analyzed with FlowJo v10 software (TreeStar).

### ASC polymerization assay (crosslinking of TCLs)

Cells were rinsed with ice-cold PBS and lysed in 20 mM HEPES, pH 7.4, 100 mM NaCl, 1% NP-40, 1 mM sodium orthovanadate, supplemented with protease inhibitors, followed by shearing with a 27-gauge needle. Insoluble pellets were resuspended in PBS supplemented with 2 mM disuccinimidyl suberate (Pierce) and incubated under rotation at room temperature for 30 min. Samples were centrifuged at 2,348×*g* for 10 min at 4 °C, and crosslinked pellets and cleared cell lysates were resuspended in Laemmli sample buffer and analyzed by immunoblot for ASC.

### ASC polymerization and NLRP3 oligomerization by immunofluorescence

HEK293 cells were stably transfected with ASC-EGFP or transiently transfected for NLRP3-EGFP and low-expressing clones were selected by limited dilution to prevent spontaneous aggregation and were grown on poly-lysine-coated coverslips. THP-1 cells were differentiated using PMA (20 nM, 16 h) on coverslips, washed in PBS and rested for 48 h before treatment and stained with mouse monoclonal anti-NLRP3 and secondary anti-mouse Alexa Fluor 488-conjugated antibodies. Cells were processed as described above and ASC and NLRP3 oligomerization was quantified using Fiji and normalized to cell numbers^60^.

### NLRP3 oligomerization by blue native polyacrylamide gel electrophoresis

For blue native polyacrylamide gel electrophoresis (Invitrogen), cells were lysed in 50 mM Bis-Tris, pH 7.2, 50 mM NaCl, 10% glycerol, 0.0001% Ponceau, 1% digitonin, 2 mM Na_3_VO_4_, 1 mM sodium fluoride and 1 mM PMSF, supplemented with 1× Protease Inhibitor Cocktail (Roche) for 30 min on ice. TCLs were triturated ten times per sample and clarified by centrifugation (16,000×*g*) for 30 min at 4 °C and analyzed on blue native polyacrylamide gels (Invitrogen), transferred onto polyvinylidene fluoride membranes and analyzed for NLRP3 or NLRP11 (Flag) expression by immunoblot.

### PLA

PLA (Duolink PLA, Millipore-Sigma) was performed according to the manufacturer’s instructions. Briefly, THP-1 cells were differentiated using PMA (20 nM, 16 h) on coverslips, washed in PBS and rested for 48 h. Following treatment, cells were washed with PBS, fixed with 3.7% paraformaldehyde for 10 min at room temperature, permeabilized with 0.2% Triton X-100 for 10 min at room temperature and washed with PBS. All incubations were performed in a humidified chamber at 37 °C. Cells were blocked with Duolink Blocking Solution for 1 h, followed by incubation with primary antibodies in Duolink Antibody Diluent for 2 h, incubated with PLUS and MINUS PLA probes, washed (Buffer A) at room temperature, incubated with Ligase for 30 min, washed (Buffer A), incubated with polymerase for 100 min, washed (Buffer B) and mounted on slides using a Duolink In Situ Mounting Medium with DAPI. Cells were analyzed by confocal microscopy (Zeiss, LSM 780).

### Statistics and reproducibility

All representative results were independently repeated at least three times with similar results, and *n* indicates the number of biological replicates. Graphs were prepared in Prism 9 (GraphPad) and data are presented as mean values ± s.d. A standard two-tailed unpaired *t*-test was used for pairwise statistical analysis of all data. Values of *P* < 0.05 were considered significant (and marked by an asterisk), and *P* values are listed in the figure legends.

### Reporting Summary

Further information on research design is available in the Nature Research Reporting Summary linked to this article.

## Online content

Any methods, additional references, Nature Research reporting summaries, source data, extended data, supplementary information, acknowledgements, peer review information; details of author contributions and competing interests; and statements of data and code availability are available at 10.1038/s41590-022-01220-3.

## Supplementary information

Reporting Summary

## Figures and Tables

**Extended Data Fig. 10:**
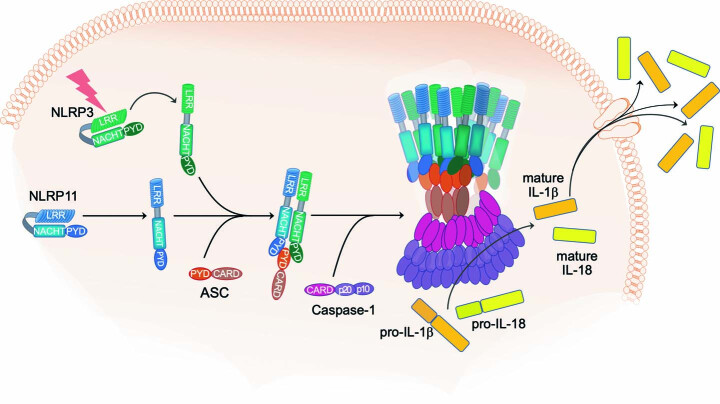
An updated model for NLRP3 inflammasome assembly and activation. Human NLRP11 binds ASC by PYRIN domain (PYD)-PYD interaction and to NLRP3 through its NACHT-LRR region, thereby acting as a scaffold for bridging ASC and NLRP3, which facilitates efficient oligomerization of NLRP3 and consequently, enables caspase-1 activation, IL-1β and IL-18 release and pyroptosis by promoting the polymerization of ASC.

## Data Availability

All data sets are provided as source data, and additional information is available from the corresponding authors upon reasonable request. Source data of intact immunoblots are included for Fig. 1, Fig. 2, Fig. 3, Fig. 4, Fig. 5, Fig. 6, Fig. 7, Extended Data Fig. 1, Extended Data Fig. 2, Extended Data Fig. 5, Extended Data Fig. 6 and Extended Data Fig. 7. Source data of graphs are included for Fig. 1, Fig. 2, Fig. 4, Fig. 5, Fig. 6, Fig. 7, Extended Data Fig. 1 and Extended Data Fig. 3. Source data are provided with this paper.

## Source data

Source Data Fig. 1 Unprocessed western blots.
Source Data Fig. 1 Statistical source data.
Source Data Fig. 2 Unprocessed western blots.
Source Data Fig. 2 Statistical source data.
Source Data Fig. 3 Unprocessed western blots.
Source Data Fig. 4 Unprocessed western blots.
Source Data Fig. 4 Statistical source data.
Source Data Fig. 5 Unprocessed western blots.
Source Data Fig. 5 Statistical source data.
Source Data Fig. 6 Unprocessed western blots.
Source Data Fig. 6 Statistical source data.
Source Data Fig. 7 Statistical source data.
Source Data Extended Data Fig. 1 Unprocessed western blots.
Source Data Extended Data Fig. 1 Statistical source data.
Source Data Extended Data Fig. 2 Unprocessed western blots.
Source Data Extended Data Fig. 3 Statistical source data.
Source Data Extended Data Fig. 5 Unprocessed western blots.
Source Data Extended Data Fig. 6 Unprocessed western blots.
Source Data Extended Data Fig. 7 Unprocessed western blots.
Source Data Extended Data Fig. 9 Unprocessed western blots.

## Extended data

Extended data are available for this paper at 10.1038/s41590-022-01220-3.
