# Supplementary material for: NLRP3 licenses NLRP11 for inflammasome activation in human macrophages
Source: Nat Immunol. 2022 May 27;23(6):892–903. doi: 10.1038/s41590-022-01220-3 (PMC9174058; doi:10.1038/s41590-022-01220-3)
Supplement: Supplementary file 1 — Reporting Summary [file 41590_2022_1220_MOESM1_ESM.pdf]

nature research

Corresponding author(s): Christian Stehlik

Last updated by author(s): Apr 7, 2022

Reporting Summary

Nature Research wishes to improve the reproducibility of the work that we publish. This form provides structure for consistency and transparency in reporting. For further information on Nature Research policies, see our Editorial Policies and the Editorial Policy Checklist.

Statistics

For all statistical analyses, confirm that the following items are present in the figure legend, table legend, main text, or Methods section.

n/a	Confirmed	
☐	☒	The exact sample size (*n*) for each experimental group/condition, given as a discrete number and unit of measurement
☐	☒	A statement on whether measurements were taken from distinct samples or whether the same sample was measured repeatedly
☐	☒	The statistical test(s) used AND whether they are one- or two-sided<br>*Only common tests should be described solely by name; describe more complex techniques in the Methods section.*
☒	☐	A description of all covariates tested
☒	☐	A description of any assumptions or corrections, such as tests of normality and adjustment for multiple comparisons
☐	☒	A full description of the statistical parameters including central tendency (e.g. means) or other basic estimates (e.g. regression coefficient) AND variation (e.g. standard deviation) or associated estimates of uncertainty (e.g. confidence intervals)
☐	☒	For null hypothesis testing, the test statistic (e.g. *F*, *t*, *r*) with confidence intervals, effect sizes, degrees of freedom and *P* value noted<br>*Give P values as exact values whenever suitable.*
☒	☐	For Bayesian analysis, information on the choice of priors and Markov chain Monte Carlo settings
☒	☐	For hierarchical and complex designs, identification of the appropriate level for tests and full reporting of outcomes
☒	☐	Estimates of effect sizes (e.g. Cohen's *d*, Pearson's *r*), indicating how they were calculated

Our web collection on statistics for biologists contains articles on many of the points above.

Software and code

Policy information about availability of computer code

Data collection	Nikon NIS Elements Advanced Research 5.2, BD FACSDiva, SKanIT 6.0.2, iBright FL1500 software version 1.7.0, Applied Biosystems QuantStudio3 1.3.3
Data analysis	Adobe Photoshop 2022, Adobe Illustrator 2022, FlowJo 10, Prism Graphpad 9

For manuscripts utilizing custom algorithms or software that are central to the research but not yet described in published literature, software must be made available to editors and reviewers. We strongly encourage code deposition in a community repository (e.g. GitHub). See the Nature Research guidelines for submitting code & software for further information.

Data

Policy information about availability of data

All manuscripts must include a data availability statement. This statement should provide the following information, where applicable:

- Accession codes, unique identifiers, or web links for publicly available datasets
- A list of figures that have associated raw data
- A description of any restrictions on data availability

Data are provided as source data and no additional data sets were collected

Field-specific reporting

Please select the one below that is the best fit for your research. If you are not sure, read the appropriate sections before making your selection.

☒ Life sciences ☐ Behavioural & social sciences ☐ Ecological, evolutionary & environmental sciences

For a reference copy of the document with all sections, see nature.com/documents/nr-reporting-summary-flat.pdf

Life sciences study design

All studies must disclose on these points even when the disclosure is negative.

Sample size	no pre-determination was performed, internal controls in each experiment with well known effect were used and samp,le size was based on prior experience
Data exclusions	no data were excluded
Replication	we replicated data at 2 different institutes; asked multiple researchers to replicate key data; used complementary approaches for testing: for example, we used siRNA, shRNA and CRISPR/Cas9 knock out to validate each approach. After pilot optimization, all representative results have been independently repeated at least 3 times with similar results and all "n" refer to biological replicates.
Randomization	no randomization and our study does not involve in vivo experiments. Cell wells were randomly assigned to each treatment group. Samples were collected and analyzed without bias using 2 researchers for sample collection and analysis, when appropriate.
Blinding	no on purpose blinding, but separating sample collection and data collection, when possible and appropriate for ELISA samples.

Reporting for specific materials, systems and methods

We require information from authors about some types of materials, experimental systems and methods used in many studies. Here, indicate whether each material, system or method listed is relevant to your study. If you are not sure if a list item applies to your research, read the appropriate section before selecting a response.

Materials & experimental systems

n/a	Involved in the study	
☐	☒	Antibodies
☐	☒	Eukaryotic cell lines
☒	☐	Palaeontology and archaeology
☒	☐	Animals and other organisms
☐	☒	Human research participants
☒	☐	Clinical data
☒	☐	Dual use research of concern

Methods

n/a	Involved in the study	
☒	☐	ChIP-seq
☐	☒	Flow cytometry
☒	☐	MRI-based neuroimaging

Antibodies

Antibodies used

Rabbit polyclonal anti-NLRP11 (Novus Biologicals, NBP1-92186), (1:1000)
Rabbit polyclonal anti-NLRP11 (Abcam, ab105408), (1:1000)
Rabbit polyclonal anti-NLRP11 (Sigma-Aldrich, HPA046402), (1:1000)
Mouse monoclonal anti-NLRP11 (custom), (1:1000)
Rabbit polyclonal anti-NLRP11 (custom), (1:1000)
Rabbit polyclonal anti-ASC (Adipogen, AG-25B-0006-C100, AL177), (1:1000)
Rabbit polyclonal anti-ASC (custom), (1:1000)
Mouse monoclonal anti-ASC (custom), (1:1000)
Mouse monoclonal anti-NLRP3 (Adipogen, Cryo-2), (1:1000)
Rabbit polyclonal anti-NLRP3 (Cell Signaling Technology, D4D8T), (1:1000)
Rabbit monoclonal anti-caspase-1 (Cell Signaling Technology , D7F10), (1:1000)
Rabbit monoclonal anti-cleaved caspase-1 (Cell Signaling Technology, D57A2) (1:1000)
Rabbit monoclonal anti-GSDMD (Cell Signaling Technology, L60), (1:1000)
Rabbit monoclonal anti-cleaved GSDMD (Cell Signaling Technology, E7H9G), (1:1000)
rabbit polyclonal anti-Caspase-4 (Cell Signaling Technology , 4450), (1:1000)
rabbit monoclonal anti-NEK7 (Abcam, EPR4900), (1:1000)
rabbit monoclonal anti-TGN46 (Invitrogen, JF1-024), (1:100)
rabbit polyclonal anti-Tom20 (Santa Cruz Biotechnology, FL-145,), (1:100)
Mouse monoclonal anti-cMyc (Santa Cruz Biotechnology, 9E10), (1:1000)
Mouse monoclonal anti-cMyc (Cell Signaling Technology, 9B11), (1:1000)
Mouse monoclonal anti-HA (Millipore-Sigma, F-7), (1:1000)

	Mouse monoclonal anti-FLAG (Millipore-Sigma, M-2), (1:1000)<br>Monoclonal anti-FLAG M2-Peroxidase HRP (Sigma, A8592), (1:2000)<br>Mouse monoclonal anti-tubulin (DSHB, AA12.1), (1:1000)<br>Goat anti-mouse IgG1 HRP (Santa Cruz Biotechnology, sc-2060), (1:10,000)<br>Protein A/G PLUS-Agarose (Santa-Cruz Biotechnology, sc-2003), beads<br>Anti-HA Magnetic Beads (Thermo Fisher, PI88836), beads<br>Anti-DYKDDDDK Magnetic Agarose (Thermo Fisher, A36797), beads<br>S-Protein Agarose (EMD Millipore, 69704-3), beads<br>Goat anti-Rabbit IgG (H+L) Alexa Fluor 488 (Invitrogen, A32731), (1:100)<br>Goat anti-Rabbit IgG (H+L) Alexa Fluor 546 (Invitrogen, A11035), (1:100)<br>Goat anti-Rabbit IgG (H+L) Alexa Fluor 647 (Invitrogen, A21245), (1:100)<br>Goat anti-Mouse IgG (H+L) Alexa Fluor 488 (Invitrogen, A11029), (1:100)<br>Goat anti-Mouse IgG (H+L) Alexa Fluor 546 (Invitrogen, A11030), (1:100)<br>Goat anti-Mouse IgG (H+L) Alexa Fluor 647 (Invitrogen, A21235), (1:100)<br>PLA probes Rabbit plus (Millipore-Sigma, DUO92002), (1:5)<br>PLA probes Mouse Minus (Millipore-Sigma, DUO92004), (1:5)<br>In Situ Detection Reagents Green (Millipore-Sigma, DUO92014),<br>In Situ Detection Reagents Red (Millipore-Sigma, DUO92008),<br>AlexaFluor 647 Streptavidin (Invitrogen, S21374), (1:10000)<br>Goat anti-Rabbit IgG (H+L) HRP (Invitrogen , 31460), (1:10000)<br>Goat anti-Mouse IgG (H+L) HRP (Invitrogen, A15999), (1:10000)<br>VeriBlot for IP Detection Reagent HRP (Abcam, ab131366), (1:200)
Validation	except well established antibodies (tubulin) we tested all antibodies using one of these approaches: epitope tag antibodies: using transient transfection of tagged cDNAs and controls and western blot analysis. Antibodies to inflammasome components were tested in cells with shRNA knock-down or CRISPR/Cas9 knock-out as well as using resting cells and cells with active inflammasome for testing cleaved caspase-1 and GSDMD, release of inflammasome components as well as ELISA assays.

Eukaryotic cell lines

Policy information about cell lines

Cell line source(s)	HEK293T (ATCC, CRL-3216),<br>Lenti-X HEK293 (Takara Bio, 632180),<br>THP-1 (ATCC, TIB-202),
Authentication	cell lines were directly obtained from the vendor or ATCC and those with a known genotype (stable expressing cells, knock out cells), were routinely tested by western blot for validation.
Mycoplasma contamination	we routinely test cell lines for Mycoplasma contamination, a statement is included in the methods section. Cells used for experiments tested negative for Mycoplasma.
Commonly misidentified lines<br>(See ICLAC register)	cell lines used in this study are not commonly misidentified and are not included in this database.

Human research participants

Policy information about studies involving human research participants

Population characteristics	unknown
Recruitment	as part of blood donation at the Cedars-Sinai Blood Bank or buffy coats purchased from the Red cross
Ethics oversight	The Cedars Sinai Blood Bank obtained informed consent under a protocol approved by Cedars Sinai Institutional Review Board. All samples were de-identified for research staff

Note that full information on the approval of the study protocol must also be provided in the manuscript.

Flow Cytometry

Plots

Confirm that:

☒ The axis labels state the marker and fluorochrome used (e.g. CD4-FITC).

☒ The axis scales are clearly visible. Include numbers along axes only for bottom left plot of group (a 'group' is an analysis of identical markers).

☒ All plots are contour plots with outliers or pseudocolor plots.

☒ A numerical value for number of cells or percentage (with statistics) is provided.

Methodology

Sample preparation	THP-1 cells were incubated with a cell-permeable, biotin labeled irreversible caspase-1 inhibitor substrate (YVAD-CMK, 20 uM) (AnaSpec). Cells were washed twice with cold PBS, fixed with 2% paraformaldehyde (Electron Microscopy Sciences) for 20m, washed twice with PBS, permeabilized with Cytofix/Cytoperm (BD Biosciences) for 20m at 4°C, washed twice with Perm/Wash buffer (BD Biosciences), stained with Alexa Fluor 647-conjuagted Streptavidin (Invitrogen), and washed twice with Perm/Wash buffer. Cells were then washed twice with cold autoMACS Running Buffer (Miltenyi Biotec), resuspended in autoMACS Running Buffer and analyzed
Instrument	BD LSRII
Software	BD FACSDiva
Cell population abundance	this was a simple 1 color analysis and 100% of singlets were included
Gating strategy	FSC-H vs. FSC-A density plot gating was performed to identify singlets and SSC-A vs. FSC-A used to gate on intact cells and gate boundaries were defined based on untreated cells and then AF-647+ cells quantified

☒ Tick this box to confirm that a figure exemplifying the gating strategy is provided in the Supplementary Information.
