# Supplementary figures and images for: NLRP3 licenses NLRP11 for inflammasome activation in human macrophages
Source: Nat Immunol. 2022 May 27;23(6):892–903. doi: 10.1038/s41590-022-01220-3 (PMC9174058; doi:10.1038/s41590-022-01220-3)

**Figure 1c**

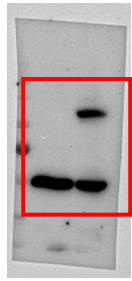

NLRP11

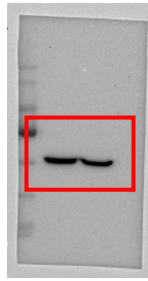

Tubulin

**Figure 1k**

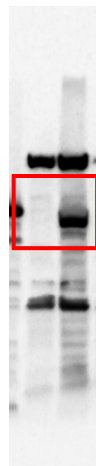

myc

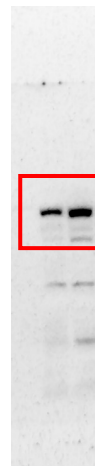

vinculin

Supplement: Source Data Fig. 1 — Unprocessed western blots. [file 41590_2022_1220_MOESM2_ESM.pdf]

Figure 2e

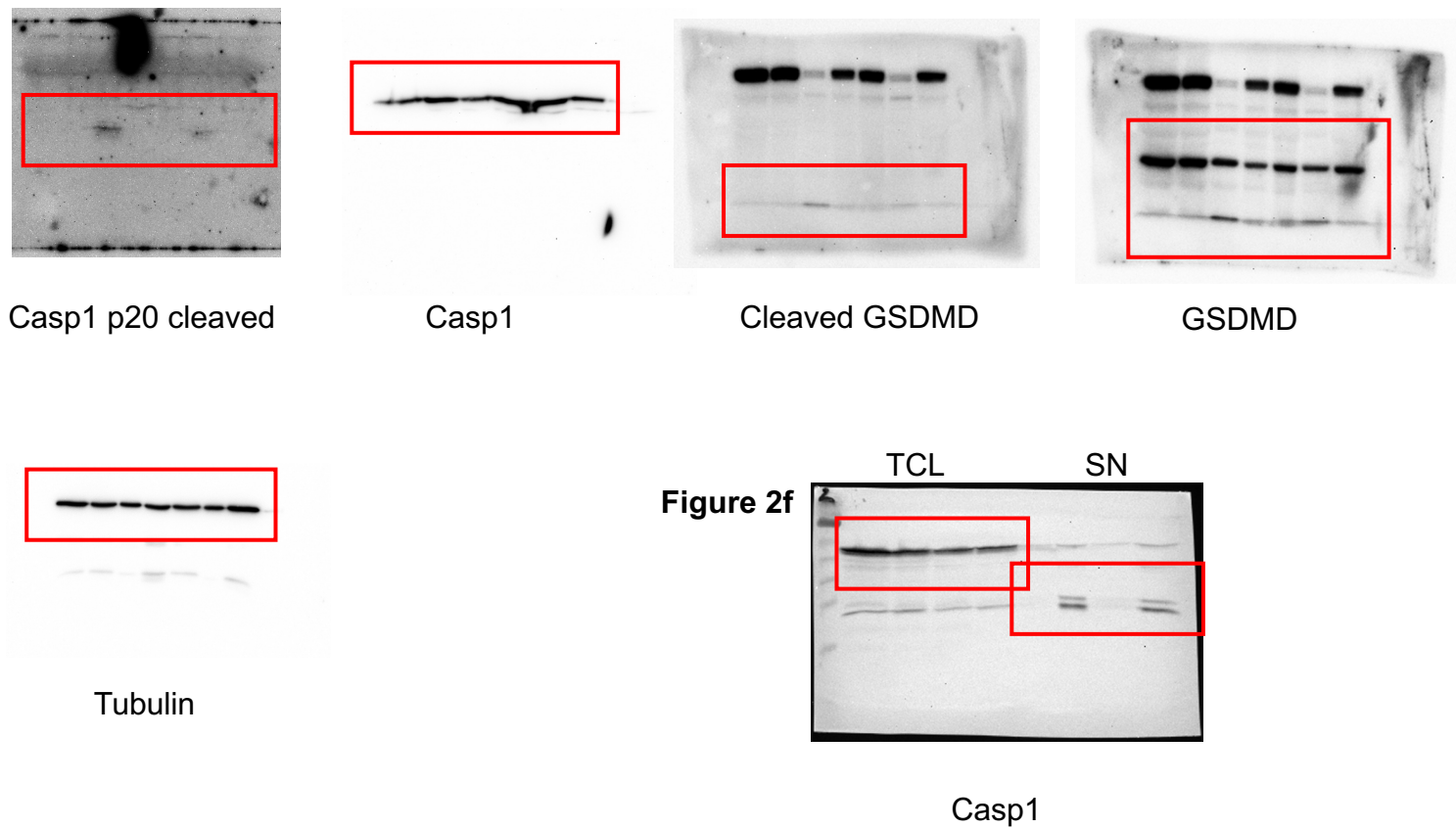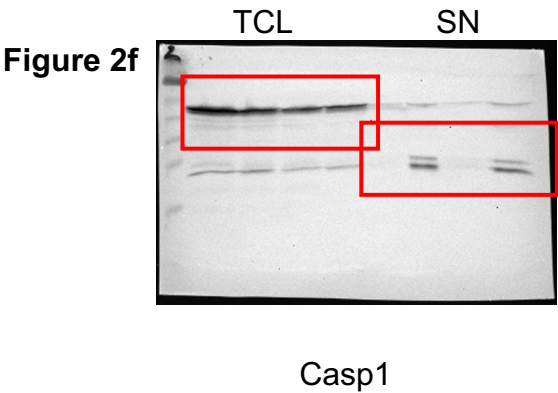

Figure 2h

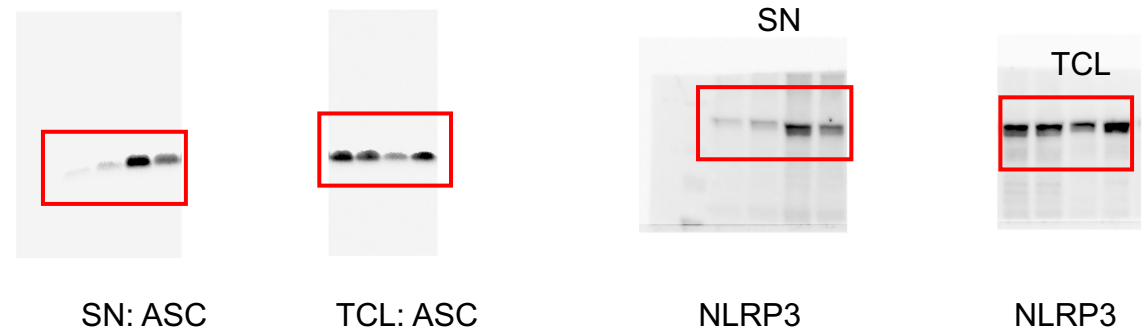

Supplement: Source Data Fig. 2 — Unprocessed western blots. [file 41590_2022_1220_MOESM4_ESM.pdf]

Figure 3c

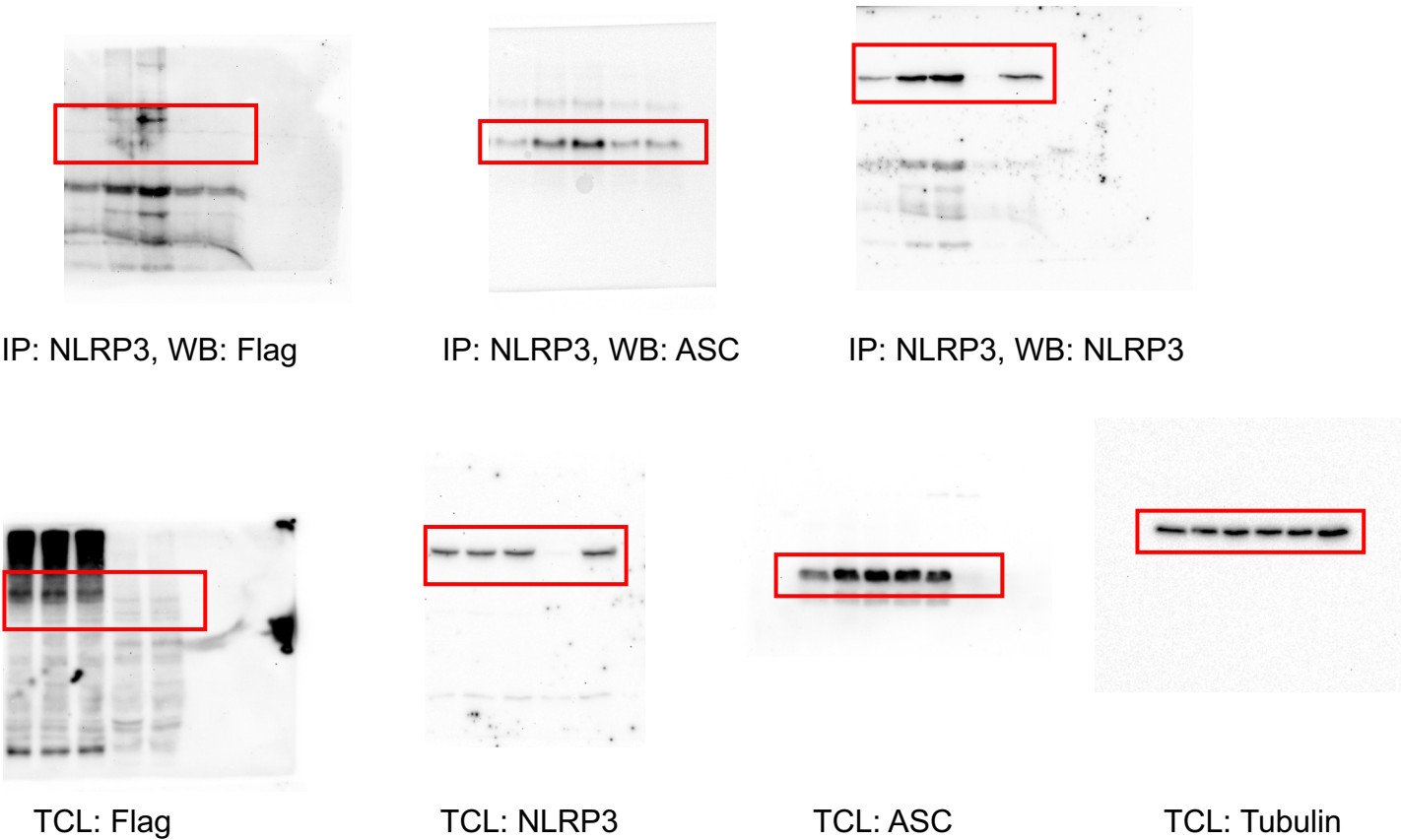

Figure 3e

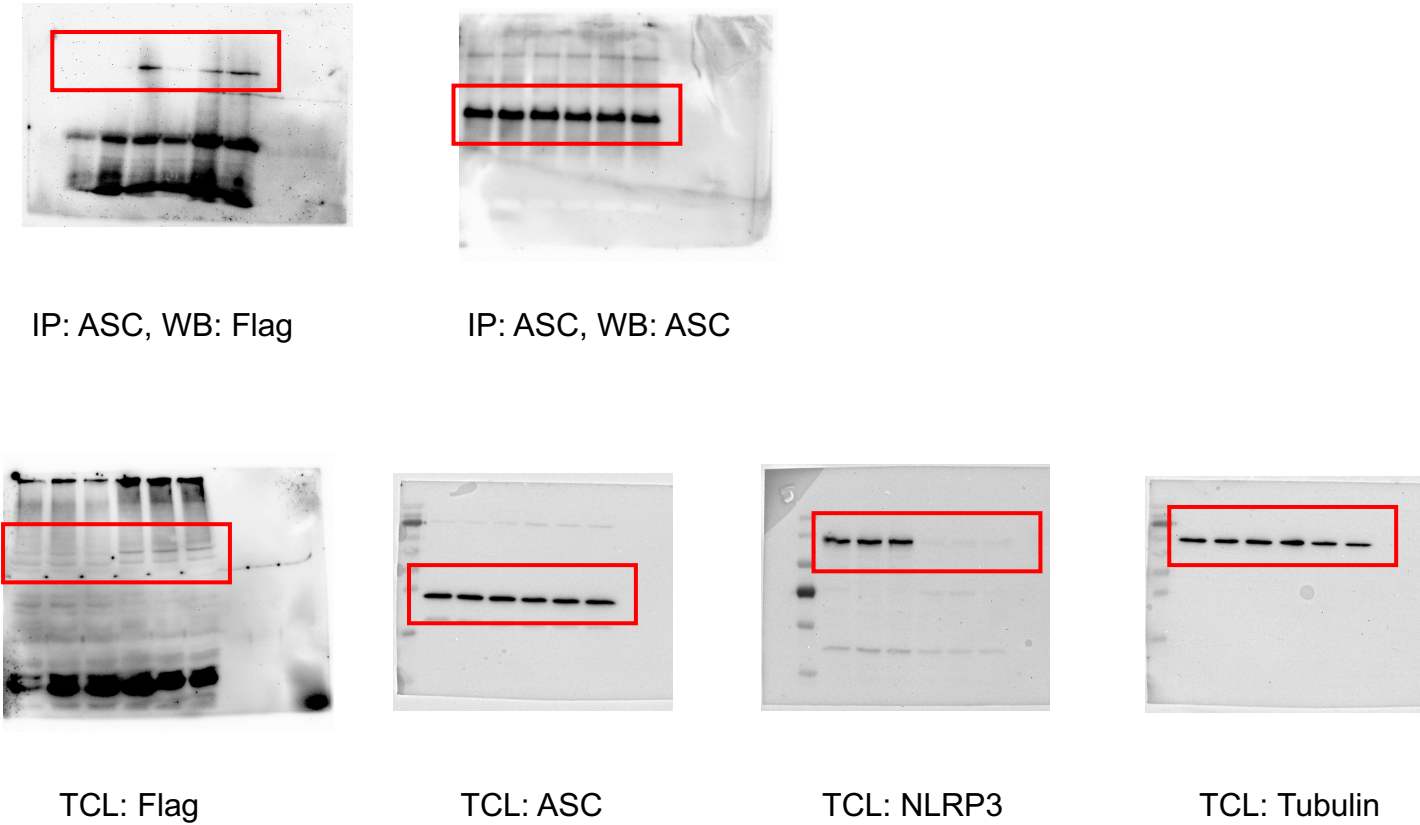

Supplement: Source Data Fig. 3 — Unprocessed western blots. [file 41590_2022_1220_MOESM6_ESM.pdf]

**Figure 5f**

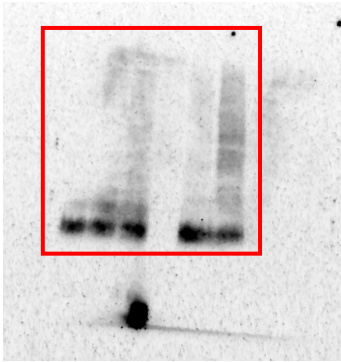

NLRP3

Flag

**Figure 5g**

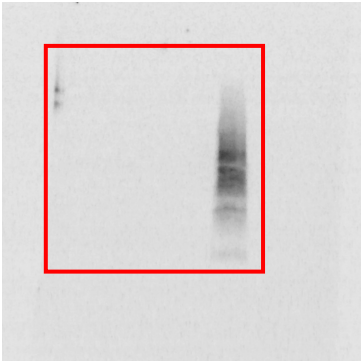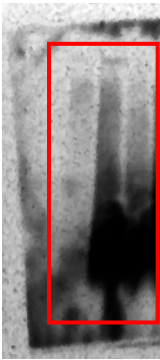

NLRP3

**Figure 5h**

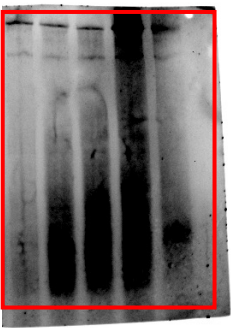

NLRP3

Supplement: Source Data Fig. 5 — Unprocessed western blots. [file 41590_2022_1220_MOESM9_ESM.pdf]

Extended Data Figure 1a

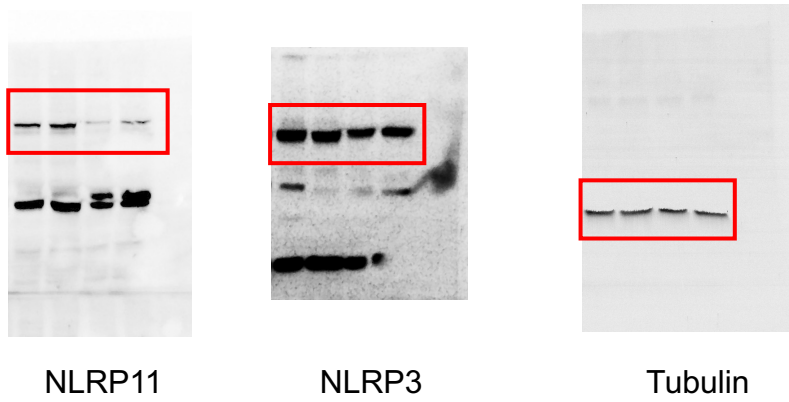

Extended Data Figure 1d

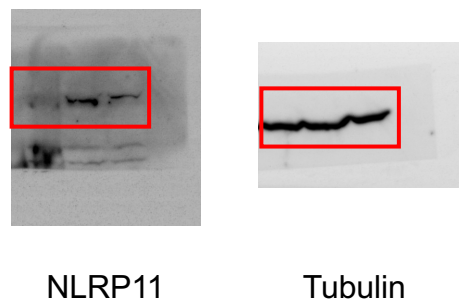

Supplement: Source Data Extended Data Fig. 1 — Statistical source data. [file 41590_2022_1220_MOESM15_ESM.pdf]

Extended Data Figure 2f

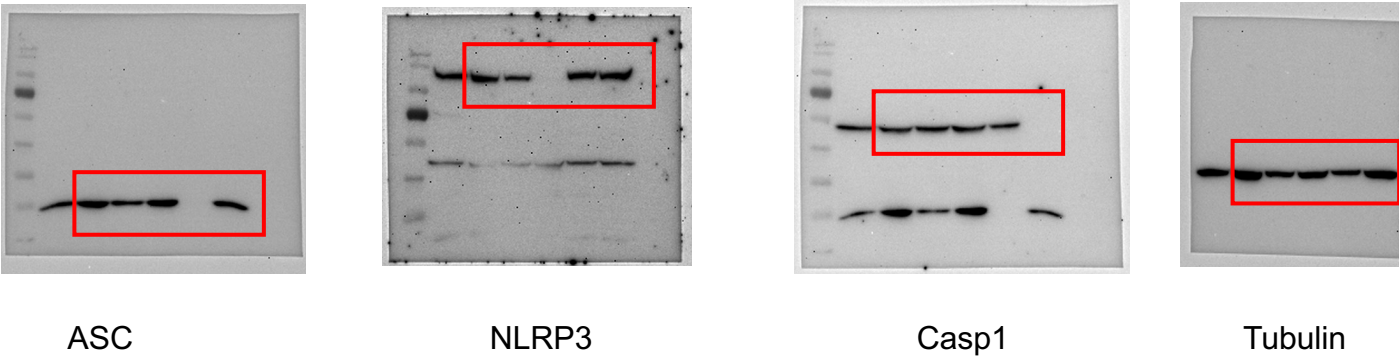

Extended Data Figure 2g

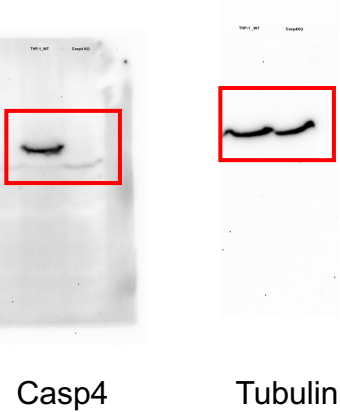

Supplement: Source Data Extended Data Fig. 2 — Unprocessed western blots. [file 41590_2022_1220_MOESM16_ESM.pdf]

Extended Data Figure 5a

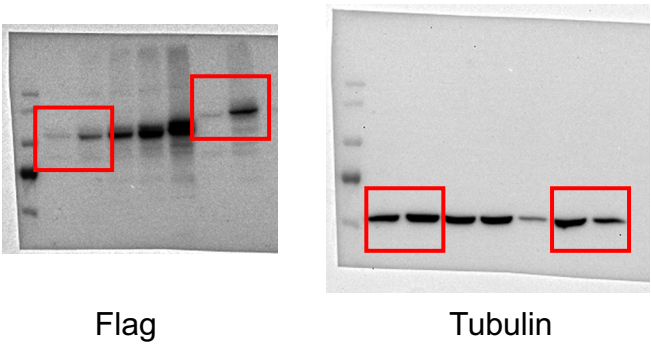

Extended Data Figure 5b

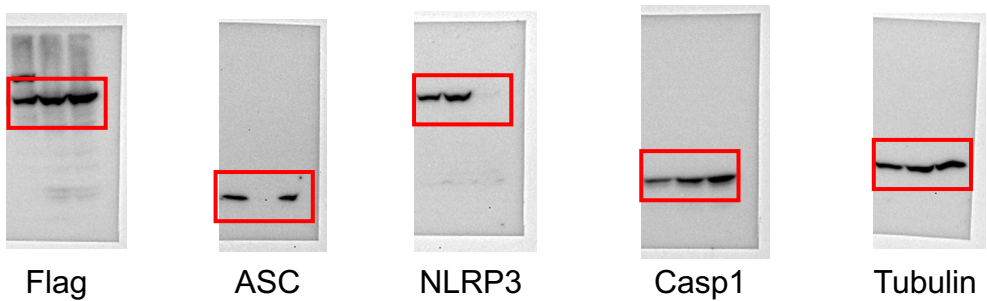

Extended Data Figure 5c

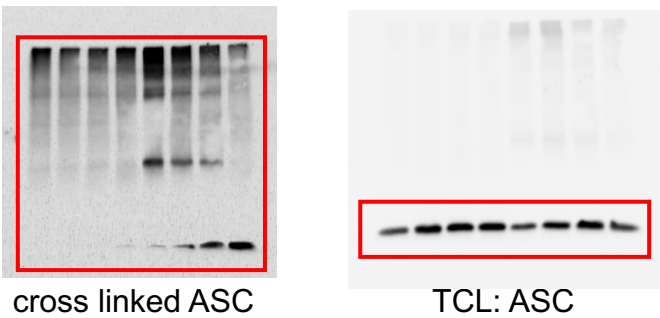

Supplement: Source Data Extended Data Fig. 5 — Unprocessed western blots. [file 41590_2022_1220_MOESM18_ESM.pdf]

Extended Data Figure 6a

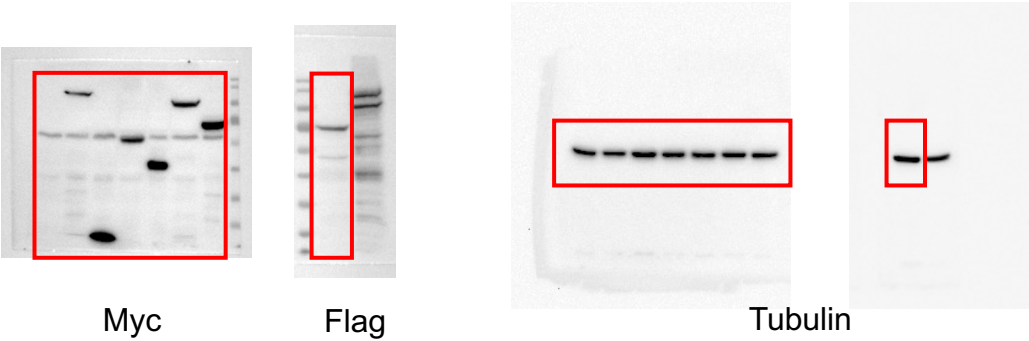

Extended Data Figure 6b

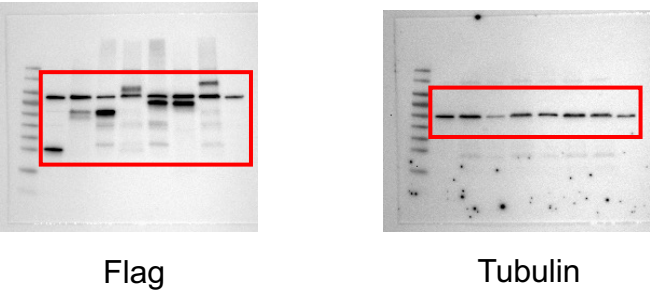

Supplement: Source Data Extended Data Fig. 6 — Unprocessed western blots. [file 41590_2022_1220_MOESM19_ESM.pdf]

Extended Data Figure 7a

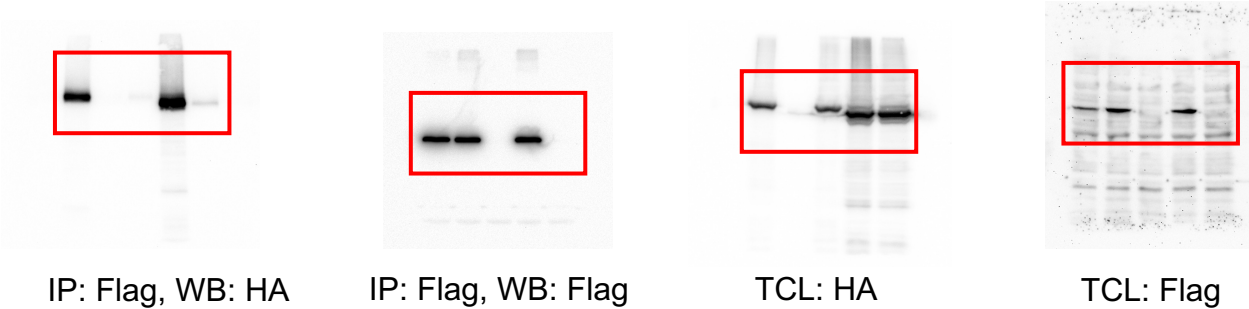

Extended Data Figure 7b

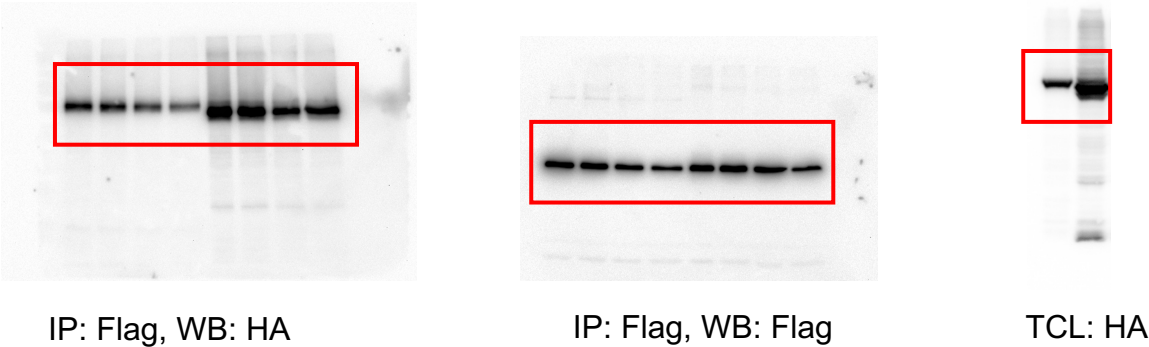

Extended Data Figure 7c

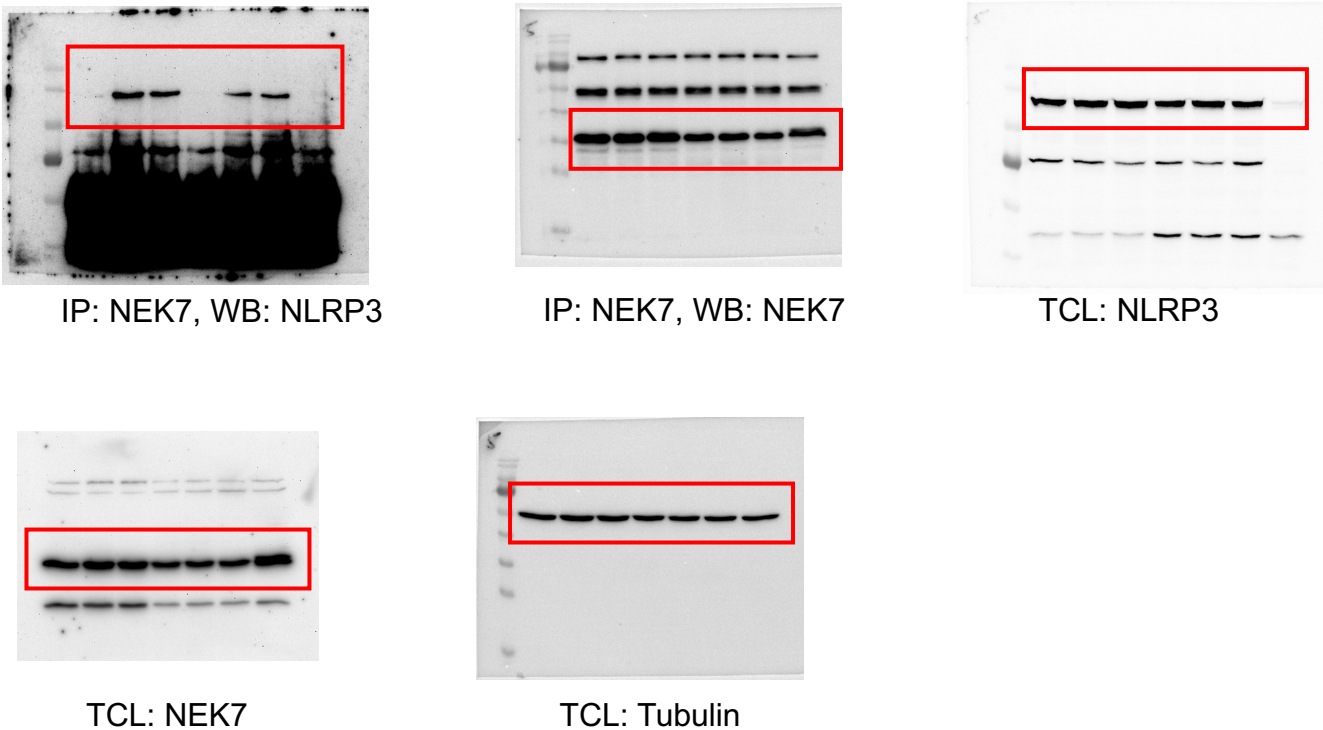

Supplement: Source Data Extended Data Fig. 7 — Unprocessed western blots. [file 41590_2022_1220_MOESM20_ESM.pdf]

Extended Data Figure 9

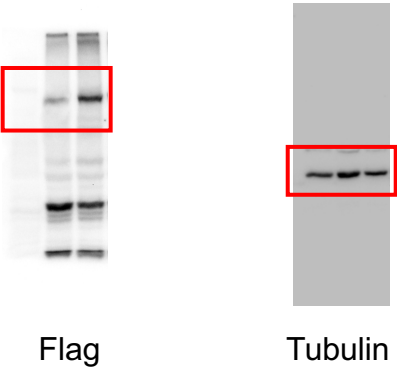

Supplement: Source Data Extended Data Fig. 9 — Unprocessed western blots. [file 41590_2022_1220_MOESM21_ESM.pdf]
