# Supplementary material for: NLRP3 licenses NLRP11 for inflammasome activation in human macrophages
Source: Nat Immunol. 2022 May 27;23(6):892–903. doi: 10.1038/s41590-022-01220-3 (PMC9174058; doi:10.1038/s41590-022-01220-3)
Supplement: Source Data Fig. 4 — Unprocessed western blots. [file 41590_2022_1220_MOESM7_ESM.pdf]

Figure 4a

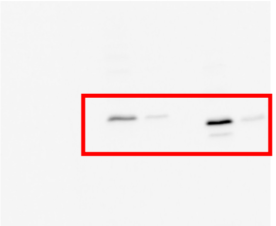

IP: Flag, WB: HA

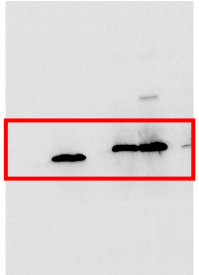

IP: Flag, WB: Flag

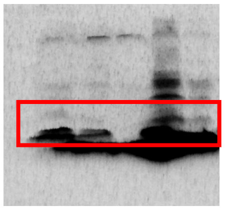

TCL: HA

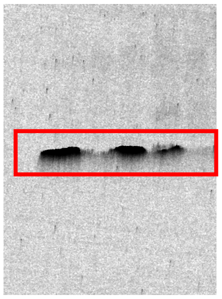

TCL: Flag

Figure 4b

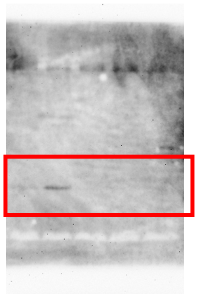

IP: HA, WB: Myc

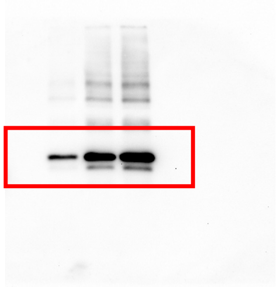

IP: HA, WB: HA

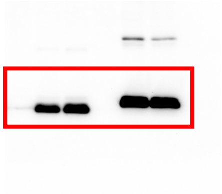

TCL: Myc

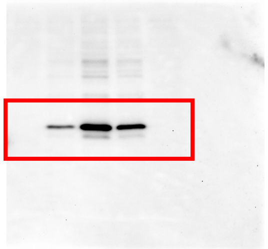

TCL: HA

Figure 4c

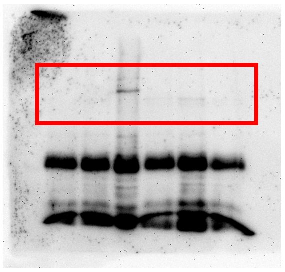

IP: ASC, WB: Flag

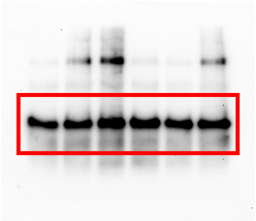

IP: ASC, WB: ASC

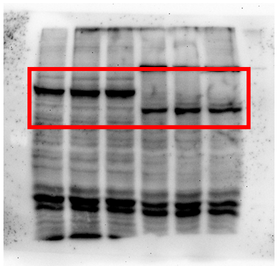

TCL: Flag

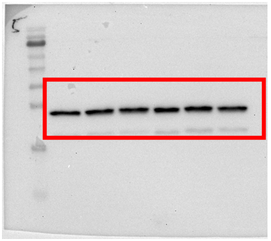

TCL: ASC

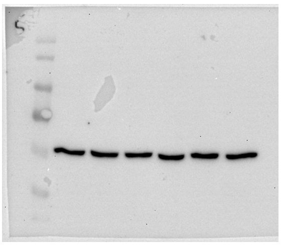

TCL: Tubulin

Figure 4f

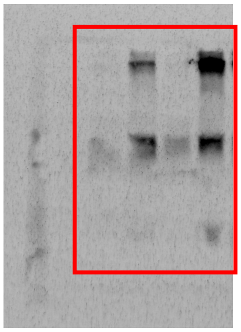

cross linked ASC

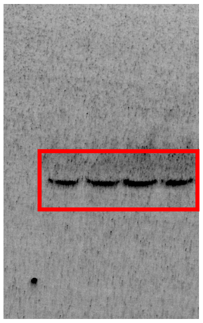

TCL: ASC

Figure 4g

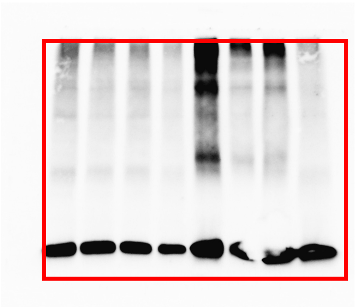

cross linked ASC

Figure 4h

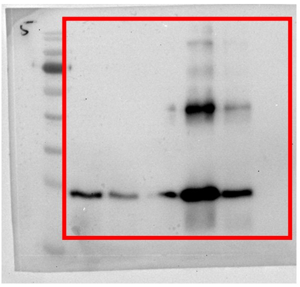

cross linked ASC

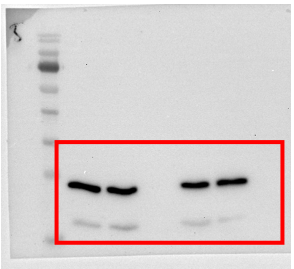

TCL: ASC
