# Supplementary material for: NLRP3 licenses NLRP11 for inflammasome activation in human macrophages
Source: Nat Immunol. 2022 May 27;23(6):892–903. doi: 10.1038/s41590-022-01220-3 (PMC9174058; doi:10.1038/s41590-022-01220-3)
Supplement: Source Data Fig. 6 — Unprocessed western blots. [file 41590_2022_1220_MOESM11_ESM.pdf]

Figure 6b

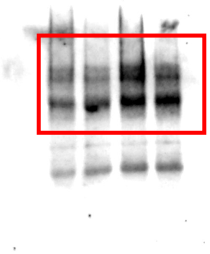

IP: NLRP3, WB: NLRP11

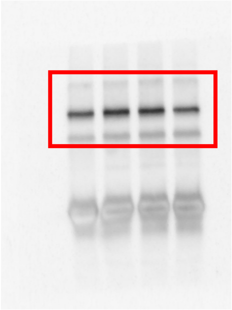

IP: NLRP3, WB: NLRP3

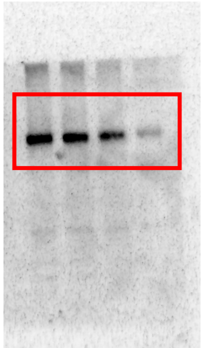

TCL: NLRP3

Figure 6c

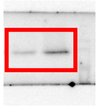

IP: Flag, WB: NLRP3 (cut membrane for large and small MW)

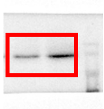

IP: Flag, WB: Flag (cut membrane for large and small MW)

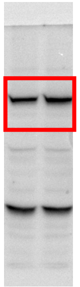

TCL: NLRP3

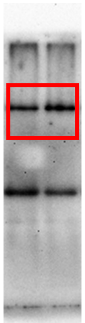

TCL: Flag

Figure 6d

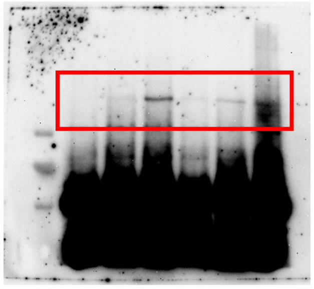

IP: NLRP3, WB: Flag

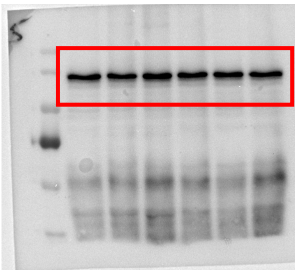

IP: NLRP3, WB: NLRP3

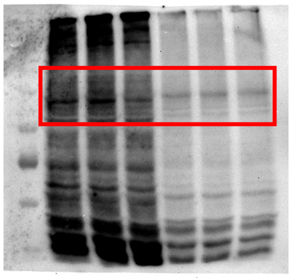

TCL: Flag

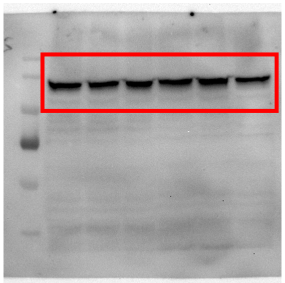

TCL: NLRP3

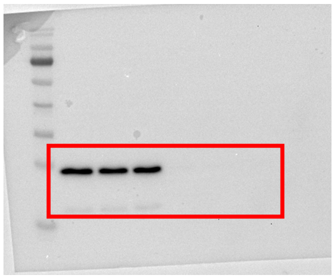

TCL: ASC

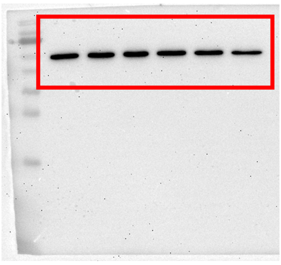

TCL: Tubulin

Figure 6e

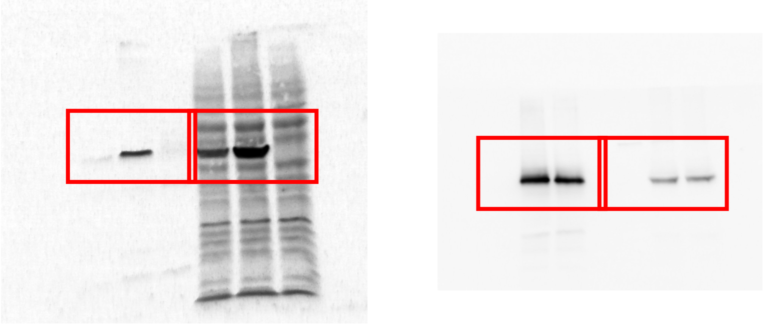

IP: HA, WB: Flag

TCL: Flag

IP: HA, WB: HA

TCL: HA

Figure 6f

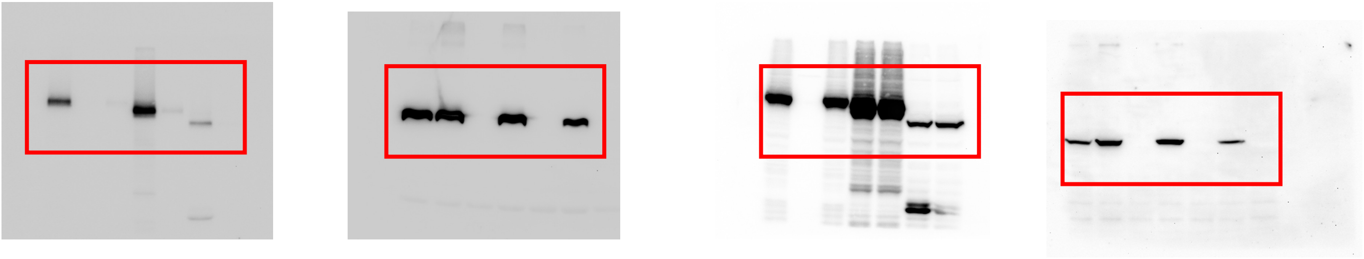

IP: Flag, WB: HA

IP: Flag, WB: Flag

TCL: HA

TCL: Flag

Figure 6g

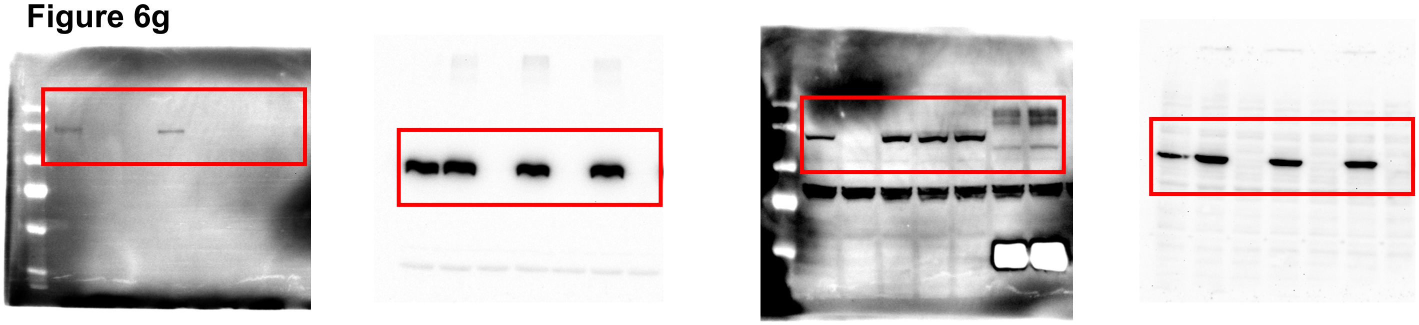

IP: Flag, WB: Myc

IP: Flag, WB: Flag

TCL: Myc

TCL: Flag

Figure 6h

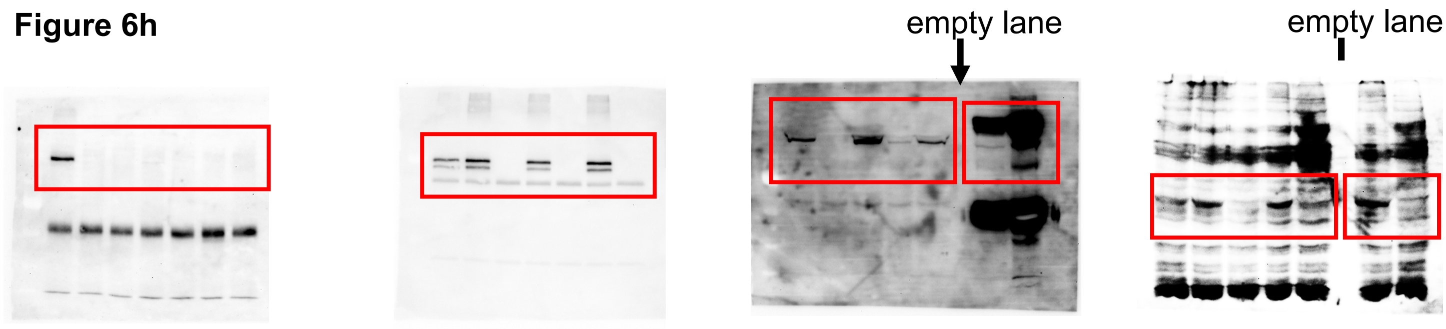

IP: GFP, WB: Myc | IP: GFP, WB: GFP | TCL: Myc | TCL: GFP

Figure 6i

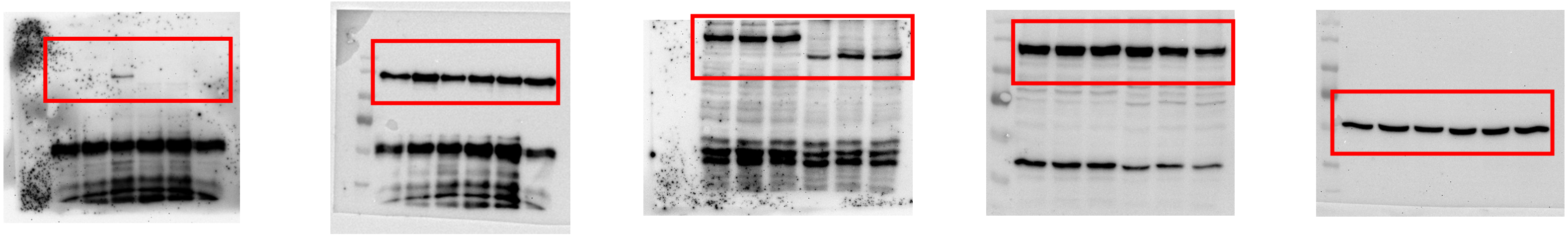

IP: NLRP3, WB: Flag | IP: NLRP3, WB: NLRP3 | TCL: Flag | TCL: NLRP3 | TCL: Tubulin
